# Malignant B-cell States Orchestrate Spatially Organized Immune Ecosystems in B-cell Lymphomas

**DOI:** 10.21203/rs.3.rs-10435263/v1

**Published:** 2026-08-03

**Authors:** Patrizia Mondello, Alisa Sadekova, ksenia fede, Surendra Dasari, Wesley Au, Moritz Binder, Kirill Kriukov, Mark Meerson, Joseph Novak, Zhi-Zhang Yang, Brianna Negaard, Ekaterina Postovalova, Jose Villasboas, Daniil Wiebe, Anastasiia Shvyrkova, Emmanuel Contreras Guzman, Nikita Kotlov, Sargis Margaryan, Alexander Nesmelov, Aleksander Sarachakov, Arman Petrosyants, Alexander Bagaev, Yucai Wang, Anne Novak, Carla Casulo, Thomas Habermann, Eric Hsi, Matthew Maurer, James Cerhan, Nathan Fowler, Rebecca King, Mark Shlomchik, Harinder Singh, Philippe Armand, Laura Pasqualucci, Richard Burack, Stephen Ansell

**Affiliations:** Mayo Clinic; Bostongene; Bostongene; Mayo Clinic; Mayo Clinic; Mayo Clinic; Bostongene; Bostongene; Mayo Clinic; Mayo Clinic; Mayo Clinic; BostonGene; Mayo Clinic; Bostongene; Bostongene; Mayo Clinic; BostonGene Corporation; Bostongene; Bostongene; Bostongene; Bostongene; BostonGene Corporation; Mayo Clinic; Division of Hematology, Mayo Clinic, Rochester, MN; Wilmot Cancer Institute; Mayo Clinic; Mayo Clinic; Mayo Clinic; Mayo Clinic College of Medicine and Science; BostonGene; Mayo Clinic; University of Pittsburgh; University of Pittsburgh School of Medicine; Mayo Clinic; Columbia University; Wilmot Cancer Institute; Mayo Clinic

**Keywords:** Tumor microenvironment, follicular lymphoma, diffuse large B cell lymphoma, Memory B cells, dark zone cells

## Abstract

Germinal center (GC)-derived lymphomas arise within a specialized immune ecosystem, yet whether malignant cells direct its remodeling remains unclear. Integrating genomic, transcriptomic, spatial, and functional analyses across follicular lymphoma and diffuse large B-cell lymphoma, we define a developmental framework linking malignant B-cell differentiation state to tumor microenvironment (TME) organization. Rather than segregating by histology, lymphomas align along a shared differentiation continuum in which proliferative dark zone (DZ) states associate with immune-depleted TMEs, whereas post-GC/memory B-cell (MBC) states drive inflamed but immunosuppressed TMEs. Spatial profiling reveals collapse of GC architecture and emergence of suppressive myeloid niches along this trajectory. Single-cell lineage reconstruction uncovers a DZ-to-MBC developmental axis underlying lymphoma evolution. Genetic reprogramming *in vivo* demonstrates that malignant differentiation state is sufficient to instruct TME remodeling. Collectively, these findings establish malignant differentiation state as a causal determinant of immune architecture across GC-derived lymphomas and identify lineage-directed immune reprogramming as a potential therapeutic strategy.

## INTRODUCTION

The tumor microenvironment (TME) is increasingly recognized as a pivotal regulator of cancer initiation, progression, and persistence.^1,2^ Far from being a passive scaffold, the TME represents a complex and dynamic ecosystem composed of immune and stromal cells, extracellular matrix components, and soluble factors that collectively shape tumor behavior.^3,4^ Through reciprocal interactions with malignant cells, the TME modulates oncogenic programs, promotes immune evasion, and contributes to disease heterogeneity and therapeutic resistance.^5-8^ Understanding the tumor-immune crosstalk has therefore become essential for elucidating the pathogenesis of both solid and hematologic malignancies.

A parallel microenvironmental paradigm governs the germinal center (GC), a transient and highly organized structure that forms within secondary lymphoid organs during T cell-dependent immune responses.^9^ In the GC, B cells undergo iterative cycles of proliferation, somatic hypermutation, and affinity-based selection across spatially and functionally distinct dark zone (DZ) and light zone (LZ) compartments. These processes rely on tightly regulated interactions among B cells, T follicular helper (Tfh) cells, and follicular dendritic cells (FDCs), which together form a specialized niche that balances clonal expansion with immune tolerance.^9^ This inherent plasticity and reliance on microenvironmental cues make GC B cells particularly susceptible to malignant transformation.

GC-derived lymphomas, including follicular lymphoma (FL) and diffuse large B-cell lymphoma (DLBCL), originate from GC B cells that acquire genetic and epigenetic alterations enabling them to hijack and reprogram this microenvironmental circuitry.^10^ Despite a shared cell-of-origin, these malignancies display striking clinical differences, ranging from the indolent course of FL to the aggressive behavior of DLBCL.^11^ While intrinsic genetic features contribute to this diversity,^12-14^ accumulating evidence suggests that TME composition, functional state, and spatial organization are key determinants of disease evolution^7,8,15-17^. In particular, disruption of antigen presentation, attenuation of immune surveillance, and emergence of immunosuppressive niches may collectively enable immune escape and sustained malignant proliferation. However, the mechanisms by which normal GC microenvironment is rewired during lymphomagenesis and how malignant B cells and their microenvironment co-evolve remain incompletely understood.

Recent advances in single-cell and spatial profiling have begun to uncover the complexity of lymphoma ecosystems^7,15,16,18^, however, a unifying framework linking malignant B-cell states, immune composition, and spatial organization across the FL-DLBCL spectrum is lacking. In particular, whether tumor-intrinsic differentiation programs actively instruct, rather than passively reflect, microenvironmental states remain unresolved. Moreover, it remains unclear whether FL3B represents a distinct biological entity or an intermediate state along the trajectory of histologic transformation. Here, we address these gaps using an integrated multi-omics approach combining bulk and single-cell transcriptomics, spatial proteomics, and genomic profiling in a prospective cohort of newly diagnosed FL and DLBCL patients. This framework enables high-resolution reconstruction of the cellular, spatial and molecular architecture of GC-derived lymphomas, providing a system-level view of tumor-immune interactions across disease evolution.

## RESULTS

### Conserved Immune-Enriched and Immune-Depleted TMEs across FL and DLBCL reflect malignant B-cell states.

**Figure 1:**
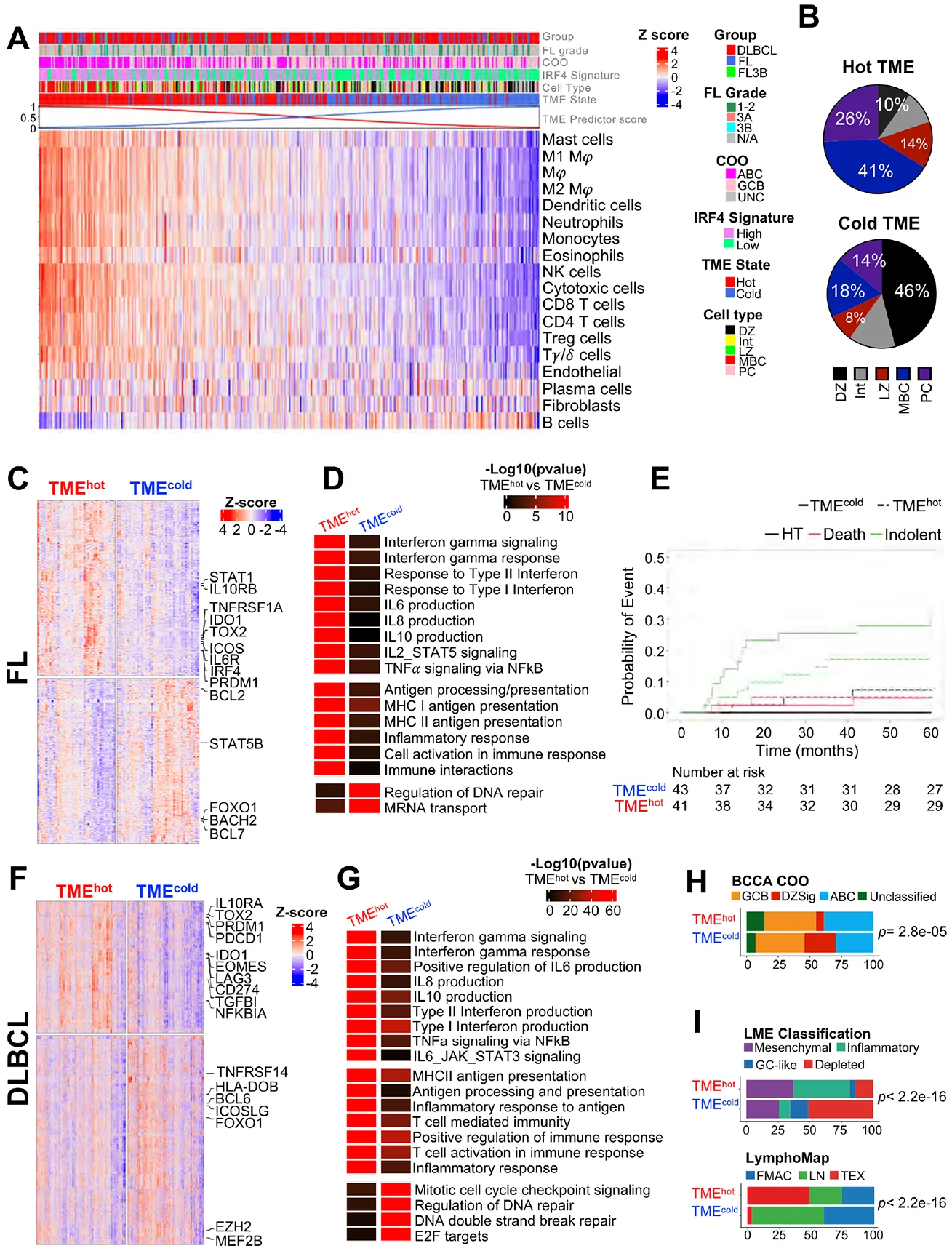
Transcriptional profiling Reveals Distinct Tumor Microenvironment States with Divergent Cell Fate Trajectories. **A.** Heatmap of immune-related gene signatures derived from RNA-seq of newly diagnosed FL (n= 86) and DLBCL (n= 304), stratified by TME status (TME^hot^ vs. TME^cold^). **B.** Prevalence of GCB signatures in TME^hot^ and TME^cold^ subgroups. **C.** Heatmap of differentially expressed genes (DEGs) from RNA-seq of sorted CD19^+^ B cells isolated from diagnostic lymph node biopsy of patients with FL (n= 86), grouped by TME status. **D.** Gene set enrichment analysis (GSEA) of DEGs shown in (**C**), highlighting significantly enriched immune signaling, antigen presentation, and cell cycle pathways in TME^hot^ vs. TME^cold^ FL. **E.** Cumulative incidence of first relapse with indolent disease, histologic transformation (HT), or death among TME^hot^ and TME^cold^ (dashed and solid lines, respectively) FL cases. **F.** Heatmap of DEGs from bulk RNA-seq of newly diagnosed DLBCL (n=304), stratified by TME status. **G.** GSEA of DEGs in (**F**), demonstrating enrichment of immune signaling, antigen presentation, and proliferative pathways in TME^hot^ vs. TME^cold^ DLBCL. **H-I.** Distribution of molecular subtypes across TME states in DLBCL, including BCCA cell-of-origin classification (**H**), and LME and LymphoMap subtypes (**I**).

To define microenvironmental programs underlying FL and DLBCL heterogeneity, we performed TME deconvolution using CIBERSORTx^19^ on bulk RNA-seq data from a prospective cohort of newly diagnosed patients, including FL1-2 (n = 61), FL3A (n = 15), FL3B (n = 10), and DLBCL (GCB n = 157, ABC n = 147).^18,20^ Unsupervised clustering of inferred cell-type proportions across all histologies identified two major TME states (k = 2), corresponding to immune-enriched (‘hot’) and immune-depleted (‘cold’) microenvironments, defined by opposing patterns of immune infiltration. To quantify this structure, we derived a linear predictor score capturing immune enrichment along a continuous axis. This score strongly concorded with cluster assignment, supporting the robustness of the inferred TME states. Notably, these TME states were not associated with lymphoma subtype and FL grade, but instead were strongly linked to tumor-intrinsic programs, including cell-of-origin (COO, p < 0.001), IRF4 signature expression^7,21^ (p < 0.001) and GC subtype (p = 0.02; Fig. 1A-B). Immune-enriched TMEs were preferentially enriched for ABC COO (59%), high IRF4 expression (69%) and post-geminal center B cell (GCB) differentiation states, including memory B cell (MBC; 41%) and plasma cell (PC; 26%) phenotypes. In contrast, immune-depleted TMEs were enriched for GCB COO (64%), low IRF4 expression (67%) and a DZ-like phenotype (46%).

Analysis restricted to FL recapitulated this dichotomy, which remained strongly associated with IRF4 status (p < 0.001) and showed a trend toward distinct GC programs (41% MBC and 19% PC in hot TME vs 38% DZ in cold TME, p = 0.07; **Fig. S1A-B**). Immune-enriched TMEs displayed increased infiltration of most immune populations (**Suppl Table S1**), except for fibroblast (exp *β* 1.61, p = 0.072) and normal B cells (exp *β* 0.39, p < 0.001). Orthogonal validation by mass cytometry (CyTOF) in matched single-cell tumor suspensions (TME^cold^ n = 39, TME^hot^ n = 30) confirmed increased abundance of T cells (p = 0.033), monocytes (p = 0.011), and NK cells (p = 0.002) in hot TMEs, and enrichment of B cells in cold TMEs (p = 0.022; **Fig. S1C**). Consistent with an inflamed microenvironment, Tfh (p < 0.007), T effector memory (Tem, p < 0.026), and T central memory (Tcm, p < 0.010) cells in hot TME showed increased HLA-DRA expression (**Fig. S1D**). CD19^+^ B cells in hot TMEs also displayed higher expression of CCR7 (p = 0.041), HLA-DRA (p = 0.001), and BTLA (p = 0.005; **Fig. S1E**), consistent with a mature and antigen-experienced B cell phenotype that may contribute to both immune engagement and immune regulatory signaling through the suppressive HVEM-BTLA axis.

Analysis restricted to DLBCL similarly revealed a robust TME dichotomy. ABC DLBCLs were preferentially enriched for hot TMEs, whereas GCB DLBCLs largely exhibited a cold TME (p = 0.012, **Fig. S1F**). This distribution was strongly associated with IRF4 status (p < 0.001) and GC subtype (p < 0.001), with MBC-like (42% vs 13%) and PC-like (28% vs 18%) states enriched in hot TMEs, and DZ-like states enriched in cold TMEs (49% vs 8%, p < 0.001; **Fig. S1G**). Consistent with findings in FL, hot TMEs in DLBCL showed broad enrichment of immune populations (**Suppl Table S2**), alongside reduced representation of normal B cells (exp *β* =0.54, p < 0.001).

### Divergent tumor states define TME polarization along a GC differentiation axis.

To define transcriptional programs associated with TME clustering, we analyzed RNA-seq profiles of purified FL B cells from 88 patients. Differential expression analysis identified 3,160 upregulated and 5,003 downregulated genes in tumors with hot versus cold TMEs, including key immunoregulatory molecules such as *IL10RB, TNFRSF1A, IDO1, TOX2, ICOS* and *IL6R* (fold change [FC] > 1.5, FDR < 0.05; Fig. 1C). Genes associated with terminal B-cell differentiation, including *IRF4* and *PRDM1*^21,22^, were enriched in FL with hot TMEs, whereas DZ-associated regulators (e.g., *FOXO1* and *BACH2*)^23,24^ predominated in cold TMEs. Gene set enrichment analysis (GSEA) using the Molecular Signature Database (MSigDB)^25^ confirmed the enrichment of immune-related pathways in hot TMEs, including antigen presentation, IFNγ response, and cytokine production (e.g., *IL6, IL10, IL8*), whereas pathways related to cell proliferation (e.g., regulation of DNA repair and MRNA transport) were downregulated (Fig. 1D). To determine whether these TME states reflect distinct tumor-intrinsic genomic features, we performed whole exome sequencing (WES) on sorted FL B cells from 75 of these patients and further expanded this cohort to a total of 113 patients (TME^hot^ n = 52, and TME^cold^ n = 61; **Fig. S2A**). Using Fisher’s exact tests with Benjamini–Hochberg correction, we identified a significant enrichment of *POU2AF1* mutations (a key regulator of MBC differentiation^26-28^) in patients with hot TMEs (13% vs 2%, p = 0.03). Additional mutations affecting genes involved in B-cell development, including *TAF1*^29,30^ (11% vs 2%, p = 0.06), *EBF1*^31,32^ (19% vs 8%, p = 0.10), and *IKZF3*^33^ (8% vs 2%, p = 0.21) were also more frequent in hot TMEs, although they did not reach statistical significance. In contrast, tumors with cold TMEs were enriched for mutations in antigen presentation genes, including *HLA-DQB1* (8% vs 0%, p = 0.04) and *HLA-DRB1* (19% vs 6%, p = 0.04; **Fig. S2B**). We next examined clinical correlates of TME status. Baseline clinical characteristics were largely comparable between groups, except for a higher prevalence of anemia (p = 0.04) and elevated FLIPI risk score (p = 0.05) in patients with cold TMEs (**Suppl Table S3**). No significant differences were observed in event-free survival (EFS; HR = 1.28, 95% confidence interval [CI] 0.59–2.73) or overall survival (OS; HR = 0.98, 95% CI 0.28–3.41) between groups, likely reflecting limited statistical power. However, restriction to cases with the most robust TME signatures (z-score > 0.5 or < −0.5) revealed a survival disadvantage in patients with cold TMEs (HR = 1.04, 95% CI 0.28–3.89, p = 0.05; **Fig. S1C**). Notably, relapse patterns differed substantially by TME status. At 5 years of follow-up, 7% of patients with hot TMEs had transformed to aggressive lymphoma compared with none of those with cold TMEs. Conversely, patients with cold TMEs were more likely to experience relapse with indolent disease (30% vs 17%; Fig. 1E and **Suppl Table S4**).

In DLBCL, differential expression analysis between hot and cold TMEs revealed patterns highly concordant with those observed in FL. Hot TMEs were characterized by upregulation of immune exhaustion molecules (e.g., *IDO1, LAG3, CD274, PDCD1, TOX2*) and genes linked to terminal B-cell differentiation, including *PRDM1*, whereas cold TMEs were enriched for DZ-associated regulators (e.g., *BCL6, EZH2, FOXO1* and *MEF2B*; Fig. 1F). GSEA confirmed marked enrichment of inflammatory and immune activation programs in hot TMEs, including IFN *γ* signaling, interleukin production, and antigen presentation, whereas cold TMEs were dominated by cell cycle-associated signatures (Fig. 1G). WES analysis of 267 DLBCL cases (hot TME, n = 149; cold TME, n = 118; **Fig. S1D**) revealed that cold TMEs were enriched for mutations in *BCL2* (15% vs 6%, p = 0.015), *TP53* (25% vs 13%, p = 0.025) and *HLA-A* (4% vs 0%, p = 0.016), consistent with enhanced proliferative capacity and impaired immune recognition. Cold TMEs also showed increased frequencies of *MEF2B* mutations (14% vs 6%, p = 0.05), further supporting a DZ-like state^34^. Conversely, hot TMEs showed higher frequencies of mutations in genes linked to terminal B-cell differentiation, including *FAS* (10% vs 3%, p = 0.05), *HIST1H1C* (12% vs 5%, p = 0.05), *TBL1XR1* (11% vs 4%, p = 0.06) and *NFKBIZ* (3% vs 0%, p = 0.06; **Fig. S2E**).^35-37^

We next evaluated the relationship between TME status and established molecular DLBCL subtypes.^14,38,39^ Using DZSig-based COO classification,^14^ hot TMEs were significantly enriched in ABC and unclassified subtypes, whereas cold TMEs were enriched in the DZSig subtype (p = 2.8e-05; Fig. 1H). In contrast, no significant association was observed with LymphGen^39^ and DLBCLclass^38^, although a modest over-representation of cold TMEs was noted within the EZB/C3 genetic clusters (**Fig. S1F**). We then compared our TME clusters with previously reported DLBCL TME classifications.^15,16,40^ A strong concordance was observed with Kotlov^15^, LymphoMAPs^16^ and EcoTyper^40^ subtypes. Inflammatory and mesenchymal Kotlov subtypes (p < 2.2e-16; Fig. 1I), as well as T-exhausted (TEX) archetype (p < 2.2e-16; Fig. 1I) were enriched in hot TMEs, whereas depleted and GC-like Kotlov subtypes, along with lymph node-like (LN) and fibroblast/macrophage-rich (FMAC) archetypes predominated in cold TMEs. Consistently, EcoTyper analysis demonstrated enrichment of immunoreactive T cell states (LE4) and NK cell-enriched (LE7) ecotypes in hot TMEs, whereas stromal-high ecotypes (LE6 and LE8) were preferentially observed in cold TMEs (p < 2.2e-16; **Fig. S1G**).

Clinically, baseline characteristics were comparable between TME groups (**Suppl Table S5**). A cold TME was associated with a trend toward inferior EFS (HR = 1.35 95% CI 0.91–1.99) and OS (HR = 1.44 95% CI 0.93–2.25) (**Fig. S1H**), with a more pronounced effect observed in cases exhibiting more robust TME signatures (z-score > 0.5 or < −0.5) (HR = 1.51 95% CI 0.88–2.59, p = 0.06; **Fig. S1I**). Collectively, these data define a conserved, cross-histology TME axis tightly linked to malignant B-cell differentiation states rather than lymphoma classification, with immune-enriched TMEs reflecting a post-GC terminally differentiated, immune-engaged B-cell program, whereas immune-depleted TMEs align with a DZ-like proliferative state characterized by impaired antigen presentation and adverse outcomes.

### Single-Cell Mapping of lymphoma cell states reveals a shared DZ-to-MBC differentiation continuum across FL and DLBCL.

**Figure 2:**
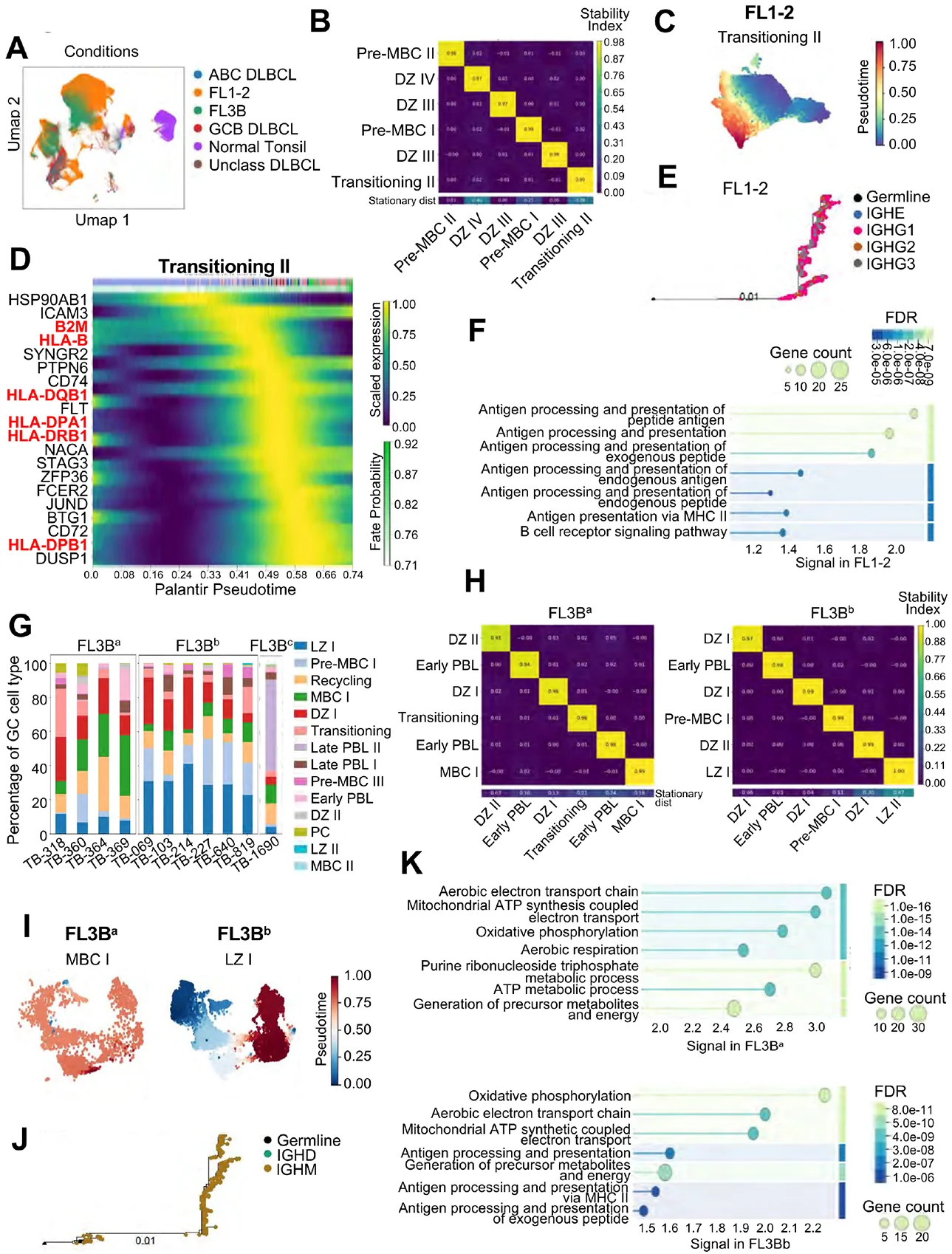
Integrative Single-Cell Mapping of Clonal Lineages and Cell-Fate Trajectories in FL. **A.** Uniform Manifold Approximation and Projection (UMAP) plot of malignant B cells from patients with FL (FL1-3A n=21, FL3B n=11) and DLBCL (GCB n=8, ABC n=3, Unclassified n=2), alongside normal germinal center B cells (GCBs) from healthy donors (n=4). Clusters are color-coded and annotated by disease subtype. **B.** Heatmap of coarse-grained transition probabilities among malignant B cell states in FL1-3A. **C.** Pseudotime ordering of FL1-3A malignant B cells inferred using the Slingshot algorithm. **D.** Smoothed expression dynamics of the top 20 genes most strongly correlated with Transitioning II fate probabilities, ordered by peak expression along pseudotime. **E.** Reconstruction of the immunoglobin heavy-chain variable region lineage tree (IgTree) for FL1-3A based on somatic hypermutation patterns derived from scBCR-seq. **F.** Gene set enrichment analysis (GSEA) of differential gene expression (DEG) in FL1-3A malignant B cells relative to normal GCBs, highlighting enrichment of antigen presentation pathways. **G.** Distribution of GCB-like subtypes in FL3B, identifying subgroups enriched for memory B cell-like (MBC-like; FL3B^a^) and light zone (LZ-like; FL3B^b^) states, as well as a third group (FL3B^c^) exhibiting elevated plasma blast (PBL) signatures. **H.** Heatmaps of transition probabilities among malignant B cell states in FL3B^a^ and FL3B^b^. **I.** Slingshot-inferred pseudotime trajectories of malignant B cells in FL3B^a^ and FL3B^b^. **J.** Representative IgTree for FL3B using scBCR-seq data. **K.** GSEA of DEG in malignant B cells from FL3B^a^ (top) and FL3B^b^ (bottom) compared with normal GCBs. FL3B^a^ is enriched for mitochondrial biogenesis and oxidative phosphorylation pathways, whereas FL3B^b^ demonstrates concurrent upregulation of mitochondrial metabolism and antigen presentation programs.

The strong association between TME polarization and malignant B cell differentiation states across lymphoma subtypes prompted us to resolve cellular hierarchies and lineage trajectories underlying these conserved transcriptional programs at single-cell resolution. To this end, we performed paired single-cell RNA-seq (scRNA-seq) coupled with single-cell BCR-seq in newly diagnosed patients with FL1-3A (n = 21), FL3B (n = 11) and DLBCL (GCB n = 8, ABC n = 3, Unclassified n = 2). In parallel, GCB cells were isolated from tonsils of healthy donors (n = 4; CD3^−^/IgD^−^/CD38^+^) and analyzed using the same workflow. We sequenced a mean of 10,007 cells per sample (range, 1,556 – 20,417), retaining 503,569 cells (mean 8,834; range, 1,418 – 19,607) after quality filtration and exclusion of double expressors of B- and T-cell markers. Following batch correction, UMAP clustering^41^ identified six major cell lineages: B cells, CD4^+^ T cells, CD8^+^ T cells, macrophages, NK and plasmacytoid dendritic cells (pDC) (**Fig. S3A-B** and **Suppl Table S6**). Normal tonsils formed a distinct transcriptional cluster, clearly separated from lymphoma samples. Consistent with known transcriptional differences, FL1-2 and DLBCL samples segregated into discrete groups, whereas FL3B tumors co-clustered with both FL1-2 and GCB DLBCL (Fig. 2A), suggesting a biological dichotomy within the FL3B subtype. B cells were classified as malignant and non-malignant cells based on VDJ clonality and inferred copy-number alteration (CNA) (**Fig. S3C**). Malignant B cells were subsequently re-clustered and annotated using established mark genes as DZ, LZ, transitioning (DZ > LZ), recycling (LZ > DZ), preMBC, MBC, and early or late plasmablast (PBL). All GCB-derived states were represented across tumors, albeit with subtype-specific differences in their relative abundance (**Fig. S3D-H**). To reconstruct lineage trajectories, we applied the CellRank algorithm^42^ to each tumor type to infer initial and terminal states and their corresponding cell fate probabilities, followed by coarse-grained transition modeling into macrostates. In FL1-2, malignant B cells originated from a DZ-like state and progressed toward three terminal states: pre-MBC, transitioning and DZ cells (**Fig. S3I**). Among these, transitioning cells emerged as the dominant terminal population, exhibiting the highest stability index (SI = 0.99) and transition probability (Fig. 2B and **Fig. S3J**). Independent pseudotime analysis using Palantir confirmed a dominant DZ-to-transitioning trajectory (Fig. 2C and **Fig. S3K**). Gene expression dynamics along this trajectory revealed enrichment of antigen-presentation programs, with top correlated genes including *B2M, HLA-DQB1, HLA-DP1A, HLA-DRB1* (Fig. 2D). This transcriptional program was supported by recurrent copy-number gains on chromosome 6p, including the MHC locus (**Fig. S3L** and **Suppl Table S7**). Consistent with a post-GC phenotype, immunoglobulin (Ig) clonotype analysis demonstrated a predominance of class-switched IGHG isotype in FL1-2 tumors (Fig. 2E and **Fig. S3M**). GSEA across tumor-types confirmed a marked enrichment of antigen presentation pathways in FL1-3A relative to other subtypes (Fig. 2F).

FL3B tumors were distinct in two subgroups based on the relative prevalence of GCB-like versus post-GCB states. FL3B^a^ tumors were enriched for MBC-like populations, whereas FL3B^b^ tumors were dominated by LZ-like cells. An additional outlier case (FL3B^c^) exhibited prominent PBL features (Fig. 2G). Trajectory inference revealed that, in both FL3B^a^ and FL3B^b^, malignant B cells originated from a common DZ-like state but diverged toward subtype-specific terminal states. In FL3B^a^, cells transitioned toward early PBL, MBC and transitioning states (**Fig. S3N**); whereas in FL3B^b^ tumors progressed toward DZ, LZ and PreMBC (**Fig. S3O**). Coarse-grained transition matrices identified the same three terminal states in each group; however, their relative dominance differed. MBC represented the predominant terminal state in FL3B^a^ (SI = 0.99), whereas LZ cells were the dominant terminal state in FL3B^b^ (SI = 1.00) (Fig. 2H). Fate probability analysis and pseudotime ordering corroborated these distinct terminal states (Fig. 2I and **Fig. S3P-Q**). Analysis of trajectory-associated gene programs revealed distinct regulatory drivers underlying these divergent fates. In FL3B^a^, *IRF8* emerged as a key regulator of the MBC-like trajectory (**Fig. S3R**). In contrast, FL3B^b^ trajectories toward the LZ state were associated with activation of *CD83* and *NFKB1*, alongside epigenetic regulators including *MEF2A, TET2, ARID1B* (**Fig. S3S**). Genomic alterations further supported this bifurcation. FL3B^a^ tumors were enriched for copy-number gains involving 1q (e.g., *MEF2D*), 11q (e.g., *CD5*) and 12q (e.g., *SMARCD1*), together with losses of 2p and 6p affecting loci such as *HLA-B/C, POU5F1, TNF*, and *NFKBI1* (**Fig. S3T**). In contrast, FL3B^b^ tumors displayed recurrent gains of 6p, involving *IRF4, DUSP22, EXOC2, HLA-B/C*, and *TNF*, as well as gains on 12q (e.g., *MAP3K12*), together with losses of 2p (e.g., *EML4, SOCS5*) and 4p (e.g., *CD38*) (**Fig. S3U** and **Suppl Table S7**). Consistent with these differences, Ig clonotype analysis revealed a predominance of unswitched IGHM isotype in FL3B, in contrast to the class-switched IGHG profile observed in FL1-2 (Fig. 2J and **Fig. S3M**). GSEA further highlighted functional divergence between subgroups, with FL3B^a^ enrichment for mitochondrial biogenesis and oxidative phosphorylation pathways, whereas FL3B^b^ showed concurrent upregulation of mitochondrial and antigen presentation programs (Fig. 2K).

**Figure 3:**
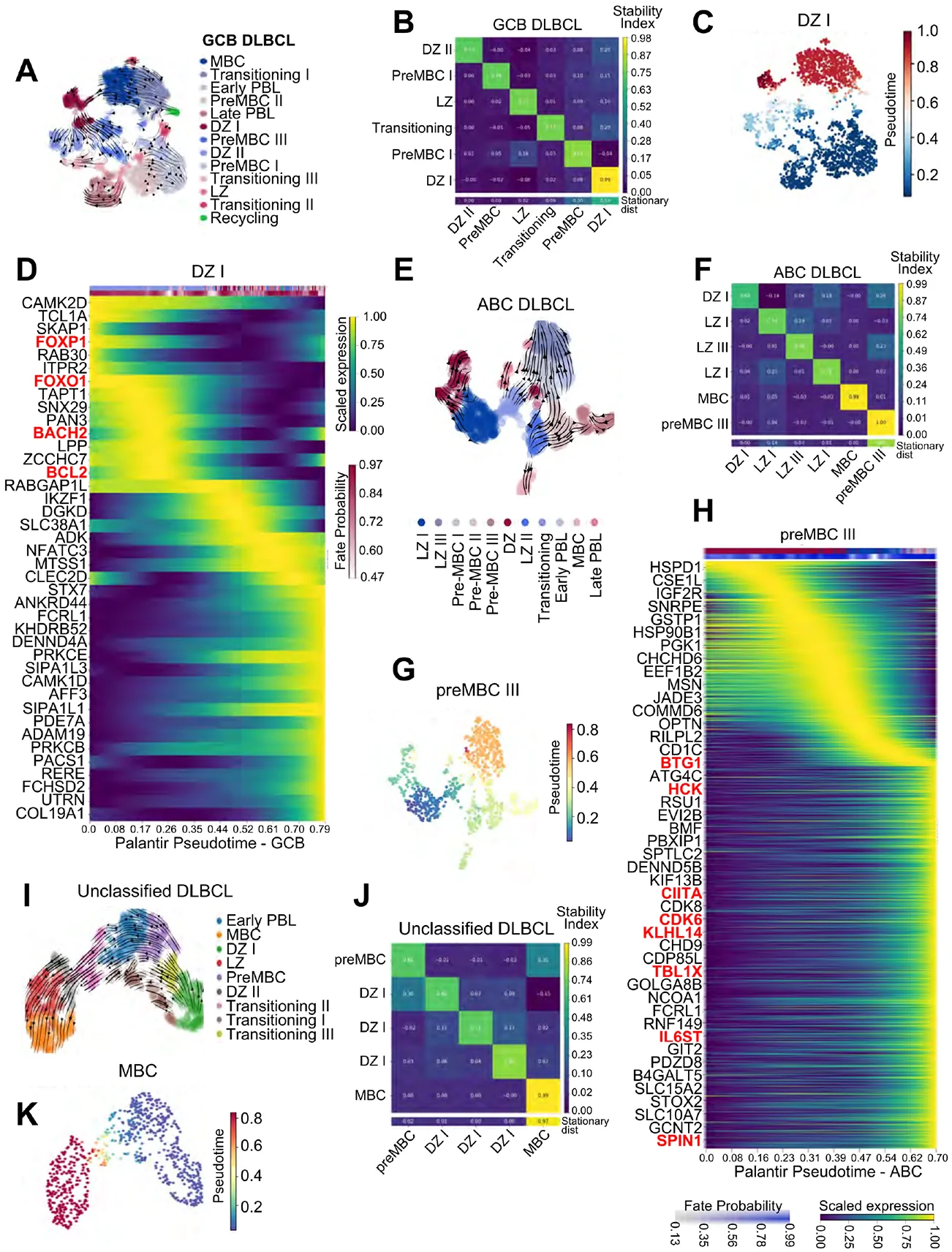
Integrative Single-Cell Mapping of Clonal Lineages and Cell-Fate Trajectories in DLBCL. **A.** RNA-velocity analysis of malignant germinal center (GCB)-like subsets in GCB DLBCL. **B.**Heatmap of coarse-grained transition probabilities among malignant B cell states in GCB DLBCL. **C.** Pseudotime trajectories of malignant B cells in GCB DLBCL inferred using the Slingshot algorithm. **D.** Smoothed expression dynamics of the top 40 genes most strongly correlated with dark zone (DZ) I probabilities, ordered by peak expression along pseudotime. **E.** RNA-velocity analysis across malignant B cell subsets in ABC DLBCL. **F.** Heatmap of coarse-grained transition probabilities among malignant B cell states in ABC DLBCL. **G.**Pseudotime trajectories of malignant B cells in ABC DLBCL. **H.** Smoothed expression dynamics of the top 50 genes most strongly correlated with pre-memory B cells (MBC) III fate probabilities, ordered by peak expression along pseudotime. **I.** RNA-velocity analysis of malignant B cell subsets in Unclassified DLBCL. **J.** Heatmap of coarse-grained transition probabilities among malignant B cell states in Unclassified DLBCL. **K.** Pseudotime trajectories of malignant B cells in Unclassified DLBCL inferred using Slingshot.

In DLBCL, ABC and GCB subtypes differed markedly in both the composition of GCB-associated cell states (**Fig. S3F-G**) and their inferred differentiation trajectories. In GCB DLBCL, malignant B cells originated from a DZ-like state and progressed toward either PreMBC or DZ, with DZ emerging as the predominant terminal state (SI 0.59) (Fig. 3A-B and **Fig. S4A**). Both fate probability analysis and independent pseudotime reconstruction consistently identified DZ-like cells as the dominant terminal population (Fig. 3C and **Fig. S4B**). Trajectory-associated gene programs were enriched for canonical GC regulators, including *FOXP1, FOXO1, BACH2*, and *BCL2* (Fig. 3D). GSEA revealed strong activation of proliferative programs, including cell cycle mitotic (NES 2.76, FDR 4.6 x 10^e–09^) and cell cycle checkpoints pathways (NES 2.88, FDR 4.6 x 10^e–09^), coupled with suppression of inflammatory signaling, including interleukin signaling (NES – 1.85, FDR 1.2 x 10^e–06^) and inflammasomes (NES – 2.15, FDR 3.5 x 10^e–05^). In contrast, ABC DLBCL tumors, while also originating from a DZ-like state, diverged toward LZ I, LZ II or preMBC (Fig. 3E and **Fig. S4C**), with preMBC representing the dominant terminal state (SI 0.82; Fig. 3F). This was consistently supported by both fate probability and pseudotime analyses (Fig. 3G and **Fig. S4D**). Trajectory-associated genes in ABC DLBCL included *BTG1, TBL1X, KLHL14, SPIN1* (Fig. 3H). Similar to GCB DLBCL, ABC tumors exhibited enrichment of proliferation programs (e.g., cell cycle mitotic [NES 1.86, FDR 1.3 x 10^e–07^], cell cycle checkpoints [NES 1.94, FDR 4.4 x 10^e–06^]), but additionally showed suppression of extracellular matrix organization (NES – 1.59, FDR 0.020) and chemokine receptor signaling (NES – 1.88, FDR 0.031). Notably, ABC DLBCL displayed a substantially higher burden of chromosomal alterations compared to GCB DLBCL, with widespread gains affecting approximately half of genome and recurrent losses, particularly on chromosome 6 (**Fig. S4E** and **Suppl Table 7**). Unclassified DLBCL most closely resembled ABC DLBCL, with malignant B cells converging toward MBC-like terminal state (Fig. 3I-J and **Fig. S4F-G**). Key regulators associated with this trajectory included *BCL2, ETV6, CCNL1, TCF4* and *ARID1B* (**Fig. S4H**). However, unclassified tumors were distinguished by upregulation of interleukin signaling (NES 1.61, FDR 0.022), chemokines signaling (NES 2.02, 0.022), and peptide ligand-binding receptor pathway (NES 1.98, FDR 0.022), alongside exhibiting structural alterations largely overlapping with those observed in ABC DLBCL (**Fig. S4I** and **Suppl Table S7**).

Together, these lineage analyses place FL1-3A, FL3B, and DLBCL along a shared DZ-to-MBC differentiation continuum. While histology influences the probability of specific terminal fates, malignant B-cell intrinsic programs ultimately determine trajectory endpoints, which in turn align with either immune-enriched or immune-depleted TMEs.

### Malignant B-cell differentiation states define distinct immune microenvironments in lymphoma.

**Figure 4:**
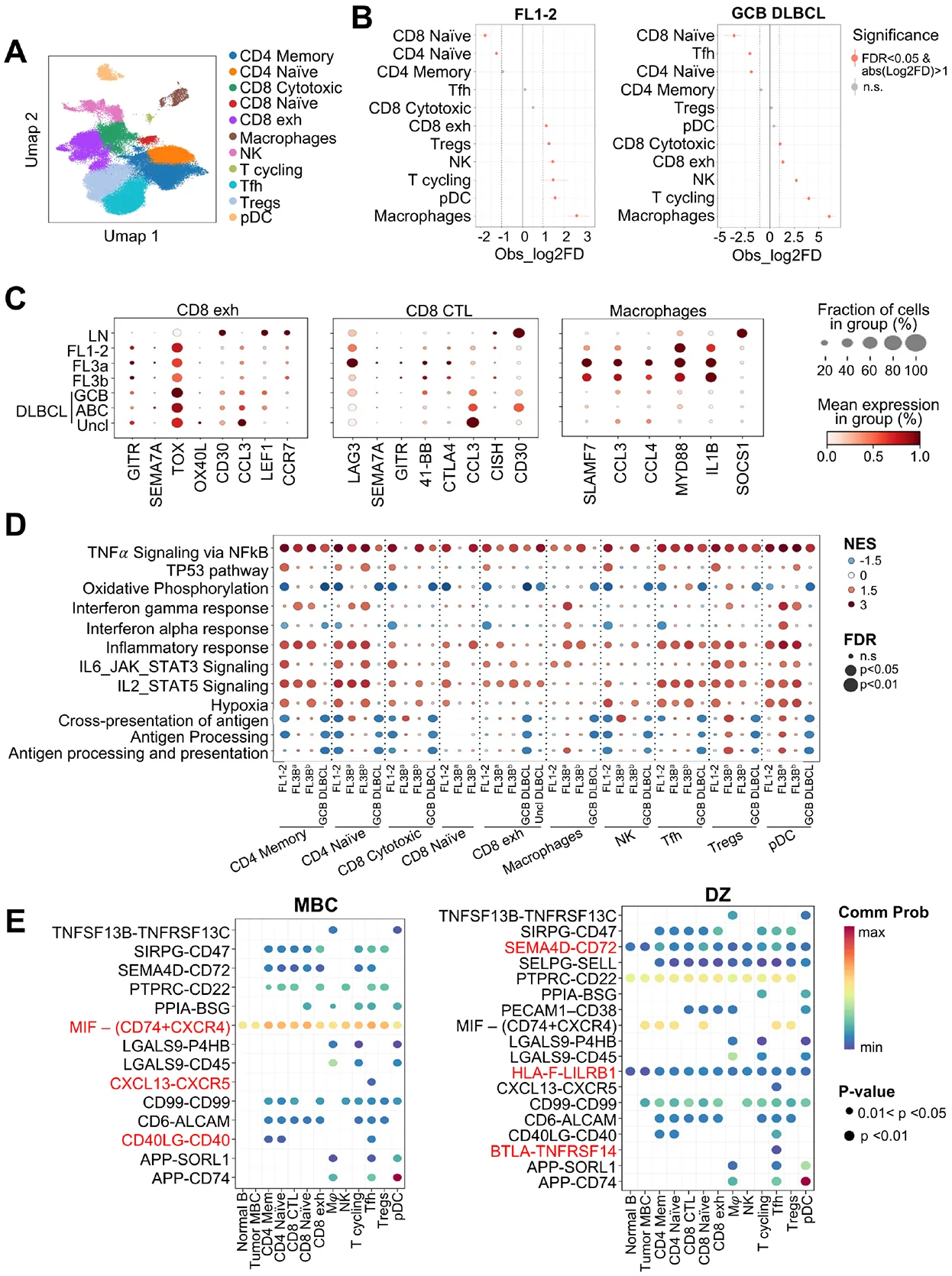
Single-cell profiling uncovers pervasive immune dysregulation and altered antigen presentation in the lymphoma TME. **A.** Uniform Manifold Approximation and Projection (UMAP) plot of immune cells from patients with FL (FL1-3A n=21, FL3B n=11) and DLBCL (GCB n=8, ABC n=3, Unclassified n=2), alongside normal lymph node tissues from healthy donors (n=4). Clusters are color-coded and annotated by immune cell subtype. **B.** Relative abundance of immune cell populations in FL1-2 (left) and GCB DLBCL (right) tumors compared with lymph nodes from healthy donors. **C.** Bubble plot of differentially expressed genes (DEG) between T-cell clusters from FL and DLBCL tumors compared with normal immune cells from healthy donors. Genes associated with immune exhaustion are annotated; circle color represents average expression, and size indicates their statistical significance. **D.** Gene set enrichment analysis (GSEA) of DEG (FC >2, FDR <0.05 in 20% cells) in immune cell clusters from FL and DLBCL relative to healthy donor immune cells. **E.**Dot plot of selected ligand/receptor interactions between T cell clusters and malignant MBC (left) and DZ cells (right) in representative FL tumors. Interaction probability is indicated by color intensity; absence of interaction is represented by the absence of dot.

Having defined conserved malignant B-cell differentiation trajectories across lymphoma subtypes, we next investigated how these tumor-intrinsic programs influence the composition and functional state of the surrounding microenvironment. We profiled immune compartments of the same 44 lymphoma samples, alongside whole lymph node tissues from healthy donors (n = 4) processed using an identical single-cell workflow. Unsupervised clustering identified 11 immune populations, annotated based on canonical marker genes^43^ as T cells, macrophages, and pDC. Subclustering of the T-cell compartment further resolved distinct functional states, including naïve (Tn), cytotoxic (CTL)-like, regulatory (Treg), Tfh, cycling and exhausted T cells (Fig. 4A and **Fig. S5A-B**). Comparative analysis revealed extensive remodeling of the lymphoma microenvironment relative to normal lymph nodes. Across both FL and DLBCL, lymphoma tissues showed consistent expansion of cycling T cells, exhausted CD8^+^ T cells, Tregs, macrophages, and NK cells, whereas CD4^+^ and CD8^+^ naïve T cells were markedly depleted (Fig. 4B and **Fig. S5C**). Notably, macrophages and NK cells were preferentially expanded in DLBCL, reaching approximately 2-fold higher abundance than in FL. Phenotypic profiling of these expanded immune populations revealed concurrent upregulation of costimulatory receptors (e.g., *GITR, SEMA7A, CD30, SLAMF7*) and immune checkpoint molecules (e.g., *LAG3, PDCD1, CTLA-4, TIGIT*; Fig. 4C and **Fig. S5D**), indicating a state of chronic immune activation coupled with functional exhaustion. Consistent with this phenotype, GSEA demonstrated broad activation of inflammatory and immune response programs accompanied by suppression of antigen presentation and metabolic pathways (Fig. 4D and **Fig. S5E**), suggesting that immune activation in lymphoma occurs with a dysfunctional immune state. Cell-cell communication analysis using CellChat^44^ further demonstrated extensive remodeling of immune interactions. Both FL and DLBCL exhibited increased MHC class I-CD8 signaling together with reduced MHC class II-CD4 interactions (**Fig. S5F-H**), indicating a reorganization of antigen-presentation networks and T-cell communication within the lymphoma TME. To determine whether malignant B-cell differentiation state shapes the TME, we compared intercellular communication networks associated with MBC-like and DZ-like malignant programs. MBC-like tumors displayed communication networks enriched for interactions linking malignant B cells with adaptive immune populations, including CXCL13-CXCR5, CD40LG-CD40, and CD6-ALCAM signaling. These pathways support lymphocyte recruitment, T-cell help, and GC organization, suggesting that MBC-like tumors preserve an immune-engaged lymphoid niche. In addition, MBC-like tumors showed prominent MIF-CD74/CXCR4 signaling, implicating malignant B-cell-driven inflammatory circuits that promote monocyte/macrophage recruitment and activation. Given the established role of MIF in shaping myeloid inflammatory niches, these findings suggest that MBC-like tumors sustain a myeloid-supported immune-inflamed microenvironment. However, this immune activation was accompanied by enrichment of LGALS9-dependent signaling (Fig. 4E), consistent with chronic immune stimulation and progressive acquisition of T-cell dysfunction. In contrast, DZ-like tumors demonstrated a fundamentally different communication architecture dominated by immune-regulatory and inhibitory signaling networks, including HLA-F-LILRB1, BTLA-TNFRSF14, and extensive SEMA4D-CD72 interactions (Fig. 4E). Despite increased overall signaling complexity, DZ-like tumors showed reduced representation of pathways associated with adaptive immune recruitment and T-cell help compared with MBC-like tumors, indicating diminished productive immune engagement. Rather than reflecting a lack of immune communication, these networks suggest that DZ-like cells actively rewire immune interactions toward suppressive and regulatory states that constrain effective antitumor immune surveillance. Collectively, these findings demonstrate that malignant B-cell differentiation state is a major determinant of lymphoma immune architecture. MBC-like tumors promote an inflamed but exhausted TME through coordinated engagement of adoptive and myeloid immune circuits, whereas DZ-like tumors favor an immune-excluded state driven by enhanced inhibitory and immune-regulatory signaling.

### Spatial profiling reveals progressive remodeling and functional reprogramming of the lymphoma microenvironment from FL to DLBCL.

**Figure 5:**
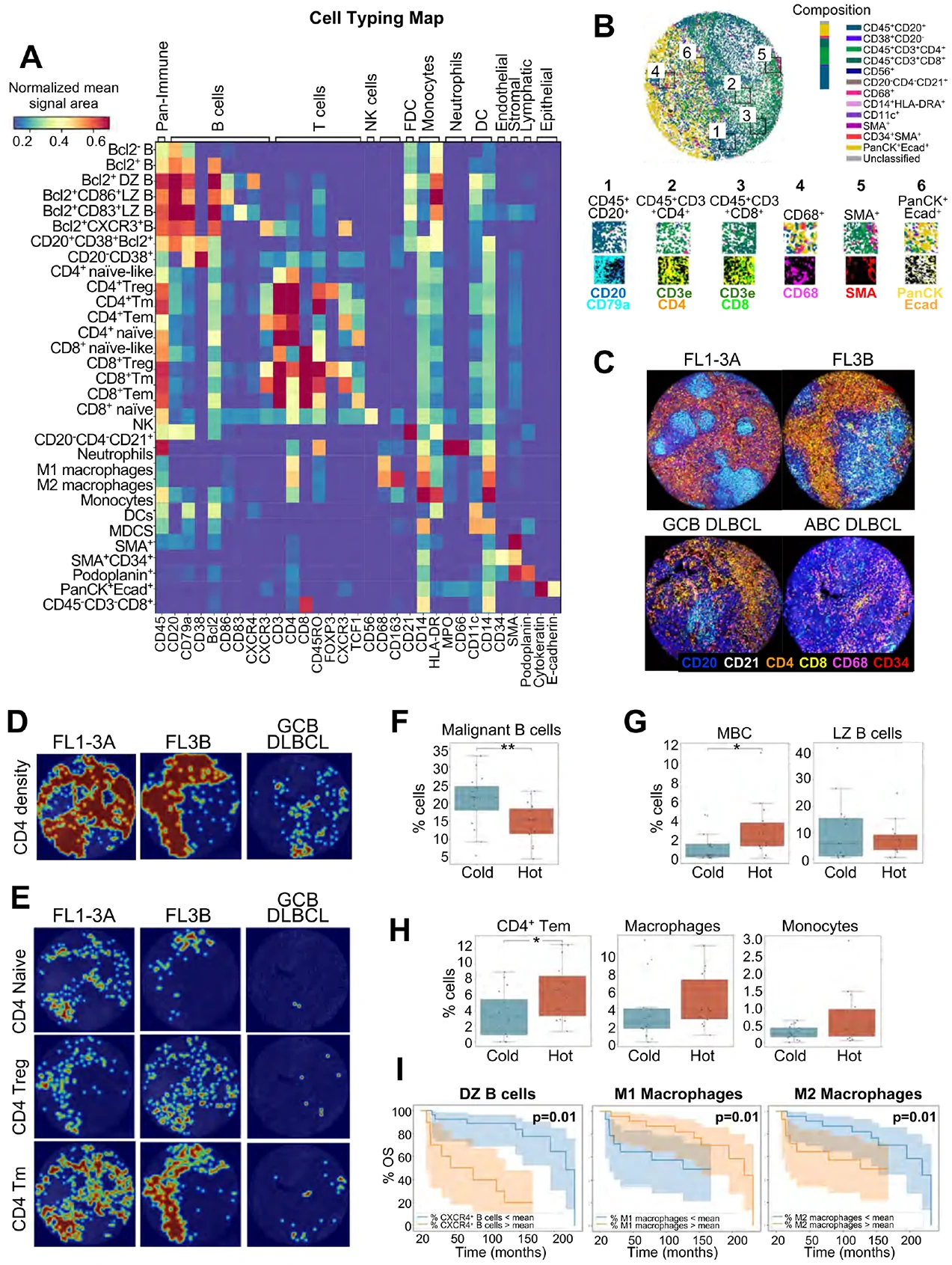
Spatial Immune Profiling reveals divergent Immune Architectures and Functional T cell states across FL and DLBCL subtypes. **A.** Heatmap of normalized mean protein marker expression across 31 cellular clusters identified by CODEX-based single-cell segmentation. **B.** Representative multiplexed CODEX images showing spatial organization and marker-defined cellular composition. **C.** Representative CODEX images of FL1-2, FL3B (top), and GCB- and ABC-type DLBCL (bottom), illustrating progressive disruption and loss of lymphoid follicular architecture. **D-E.** Representative spatial density maps showing distribution of overall CD4^+^ T cells (**D**) and CD4^+^ T cell subsets, including naïve, regulatory (Tregs) and memory (Tm) populations (**E**), across FL1-2, FL3B, and GCB DLBCL. **F-H.** Quantification of the relative abundance of the indicated B cell populations (**F-G**) and immune cell populations (**H**) in TME^hot^ vs. TME^cold^ states. Statistical significance was assessed using the Kruskal-Wallis test followed by Dunn's multiple-comparison test with Benjamini-Hochberg correction. *, P < 0.05; **, P < 0.01. **I.** Kaplan-Meier curves of overall survival in FL and DLBCL patients stratified by the expression of CXCR4 in malignant B cells, and M1 and M2 macrophages (above mean, orange; below mean, blue).

To define how these dysfunctional immune states are spatially organized within lymphoma tissues, we performed multiplex immunofluorescence (MxIF) imaging (PhenoCycler-Fusion) on tissue microarrays (TMAs) from lymph nodal tumor samples of paired FL1-3A (n = 20), FL3B (n = 9), and DLBCL (including GCB, n = 7 and ABC, n = 6). Reactive tonsil and spleen tissues from 5 healthy donors, as well as liver tissue and peripheral blood mononuclear cells (PBMCs) from two additional healthy individuals, were included as normal controls. For cell type identification and quantification, cells were segmented using a convolutional neural network and annotated based on the dominant marker expression (**Suppl Table S8**) into 14 major populations: B cells (CD45^+^CD20^+^), T cells (CD45^+^CD3^+^), macrophages (CD68^+^), monocytes (CD14^+^), NK cells (CD56^+^), plasma cells (PC, CD20^−^CD38^+^), neutrophils (CD66^+^MPO^+^), FDCs (CD20^−^CD21^+^), dendritic cells (CD11c^+^), lymphatic endothelial cells (podoplanin^+^), endothelial cells (CD34^+^), epithelial cells (Pan-CK^+^), stromal cells (SMA^+^) and splenic littoral cells (CD8^+^CD3^−^). T cells were further stratified into CD4^+^ and CD8^+^ subsets and functionally annotated into naïve (TCF1^+^), memory (Tm, CD45RO^+^), effector memory (Tem, CXCR3^+^) and regulatory (FOXP3^+^) phenotypes. CD4^+^ T cells were also distinguished in Tfh (PD1^+^) and T helper (VISTA^+^) subsets. All T cell populations were characterized by the expression of activation and exhaustion-associated markers, including IFNG, GZMB, CD44, IDO1, PD1, LAG3, TOX. Macrophages were classified as M1-like and M2-like based on CD163 expression. Additionally, myelodysplastic dendritic cells (mDC) were identified based on the co-expression of CD11c and CD14 within the dendritic cells, which in absence of HLA-DR were annotated as myeloid-derived suppressor cells (MDSCs) (Fig. 5A**-B** and **Fig. S6A**). Within the B-cell compartment, GCB-like cells were classified as LZ (CD86^+^ and/or CD83^+^) and DZ (CXCR4^+^) subsets. BCL2 expression was used to distinguish benign (CD45^+^CD20^+^BCL2^−^) from malignant (CD45^+^CD20^+^BCL2^+^) B cells. Putative tumor B cells were further characterized as MBC-like (CD20^+^BCL2^+^CXCR3^+^) and PBL-like (CD20^+^CD38^+^BCL2^+^) phenotypes (**Fig. S6B-C**).

As expected, follicular architecture was progressively disrupted from FL1-3A to FL3B and was largely absent in DLBCL (Fig. 5C). This was accompanied by a progressive decrease in the FDC network, with the lowest expression observed in ABC DLBCL (**Fig. S6D**), consistent with loss of GC organization. To characterize cell composition across tumor tissues, we performed cell density analyses which revealed distinct spatial patterns of biologically relevant immune populations. CD4^+^ T-cell density was significantly higher in FL1-3A (p = 0.001) and FL3B (p = 0048) compared with DLBCL (Fig. 5D and **Fig. S6E**), whereas CD8^+^ T-cell density did not differ significantly among these subtypes. FL1-3A tumors showed enrichment of Tfh (p = 0.001) and CD4^+^ T helper cells (p = 0.037; **Fig. S6F**), together with higher proportions of naïve (p = 0.0002), regulatory (p = 0.003) and memory (p = 0.030) CD4^+^ T cells compared with DLBCL, particularly the GCB subtype. FL3B displayed the highest proportion of Tregs across all subtypes (p = 0.001; Fig. 5E and **Fig. S6G**), indicative of increased immunoregulatory pressure in higher-grade FL. Notably, CD4^+^ Tm cells in FL3B and DLBCL exhibited increased expression of activation (GZMB^+^, p = 0.035 and p = 0.006 respectively) and exhaustion (IDO1^+^, p = 0.013) markers compared with FL1-3A (**Fig. S6H**). Effector memory CD4^+^ T cells displayed subtype-specific polarization, with higher Th1-like frequencies (CD4^+^CD44^+^, p = 0.043; CD4^+^IFNG^+^ p = 0.001) in ABC compared with GCB DLBCL (**Fig. S6I**), indicating disease-specific immune activation patterns.

Despite comparable numbers and densities of total CD8^+^ T cells across groups, CD8^+^ Tregs were more abundant in FL1-3A and FL3B than in DLBCL (p = 0.012), with no significant difference between FL grades (**Fig. S6J**). CD8^+^ Tm cell density was higher in FL1-3A and FL3B compared to ABC DLBCL (p = 0.032), but comparable to that in GCB DLBCL. However, CD8^+^ Tm in GCB DLBCL showed higher expression of GZMB than FLs (80% in GCB vs 34% in FL1-3A vs 45% in FL3B; p = 0.001) (**Fig. S6K**), suggesting a shift towards cytotoxic activation in DLBCL. Similarly, CD8^+^ T naïve cells were more abundant in FL1-3A, FL3B and ABC DLBCL than in GCB DLBCL (FL1-3A, p = 0.024; FL3B, p = 0.008; ABC DLBCL, p = 0.008), but showed higher GZMB expression in DLBCL, particularly in the GCB subtype (p = 0.039; **Fig. S6L**).

With respect to other immune populations, FL1-3A was enriched for NK cells relative to DLBCL (p = 0.016; **Fig. S6M**). In contrast, DLBCL showed higher abundance of macrophages (p = 0.002) and mDCs (ABC, p = 0.037; GCB, p = 0.002) than FL1-3A (**Fig. S6N**). Notably, mDC populations also differed between FL1-3A and FL3B, suggesting progressive myeloid remodeling during disease evolution. ABC DLBCL additionally exhibited increased neutrophil infiltration (FL3B, p = 0.005; GCB, p = 0.022; **Fig. S6O**). Collectively, these findings demonstrate progressive remodeling of the TME from FL to DLBCL, characterized by loss of follicular architecture, expansion of myeloid and suppressive compartments, and functional reprogramming of both CD4^+^ and CD8^+^ T cell states. While FL retains a spatially organized and immunologically active microenvironment, DLBCL, particularly the ABC subtype, exhibits coordinated immune reprogramming toward suppressive and dysfunctional T-cell states.

### GC state remodeling of malignant B cells underlies progressive proliferation and immune escape across FL and DLBCL.

Consistent with the indolent nature of FL, tumor B cells in FL1-3A exhibited significantly lower Ki67 expression compared with DLBCL subtypes (p = 0.0001, **Fig. S7A**). A similar proliferation pattern was observed across FL grades, with higher proportions of Ki67^+^ B cells in FL3B relative to FL1-3A (p = 0.041). We next examined the distribution of GC-derived B subsets^45^. The proportion of malignant LZ-like cells (CD20^+^CD83^+^BCL2^+^) was highest in FL1-3A and progressively decreased across FL3B and DLBCL (p = 0.020), whereas DZ-like cells (CD20^+^CXCR4^+^BCL2^+^) showed a trend toward increasing abundance from FL1-3A to DLBCL, although this did not reach statistical significance (**Fig. S7B**). Notably, both LZ and DZ populations in DLBCL showed higher expression of the immune suppressive checkpoint PD-L1 compared with FL (p < 0.05, **Fig. S7C**), accompanied by reduced HLA-A and HLA-DR expression (**Fig. S7D**), consistent with attenuation of antigen presentation capacity. No significant differences were observed in MBC-like and PBL-like populations, likely reflecting limited statistical power in these subsets. Overall, these data support a progressive shift from low-proliferative, LZ-enriched B cells in FL1-3A to highly proliferative, immune-evasive DZ B cells in DLBCL.

### Distinct tumor and immune cellular subsets define Hot and Cold TME states and their clinical impact.

The link between malignant B-cell state and TME polarization raised the possibility that specific cellular subsets structurally define hot and cold microenvironment states. We therefore examined whether distinct tumor and immune populations are differentially enriched across TME classes. Cold TMEs were markedly enriched for both benign and malignant B-cell populations (p < 0.01; Fig. 5F and **Fig. S7E**). Within the malignant compartment, MBC-like populations were preferentially associated with hot TMEs (p = 0.021), whereas LZ-like B cells were enriched in cold TMEs (Fig. 5G and **Fig. S7F**), supporting the notion that tumor B-cell differentiation state shapes TME composition and architecture. The spatial association of LZ-like cells with cold TMEs likely reflects preservation of follicular architecture in a subset of cases resembling lymph node-like organization, and should be distinguished from the DZ-like malignant transcriptional programs that characterize the broader cold-state phenotype. Regarding the immune infiltrates, hot TMEs were enriched for CD4^+^ Tem cells, monocytes, macrophages, and MDSCs (p = 0.035; Fig. 5H and **Fig. S7G**).

We next evaluated the prognostic implications of these cellular patterns. Across the overall cohort, higher proportions of benign B cells (HR = 0.95, CI 95% 0.92–0.99) and M1 macrophages (HR = 0.98, CI 95% 0.96–0.99) were associated with improved EFS, whereas increased M2 macrophage abundance correlated with inferior outcomes (HR = 1.02, 95% CI 1.00-1.03; **Fig. S7H**). Among T-cell subsets, enrichment of CD8^+^ Tn-like (FOXP3^−^CXCR3^−^TCF1^−^) cells was associated with increased risk of failing EFS24 (p = 0.037; **Fig. S7I**). Notably, higher infiltration of DZ-like cells correlated with inferior OS (HR = 1.06, CI 95% 1.01-1.11Fig. 5I). Consistently, macrophage polarization showed opposing prognostic effects, with M1 macrophage associated with improved survival (HR = 0.97, CI 95% 0.95–0.99; p = 0.016) and M2 macrophages with worse OS (HR = 1.02, CI 95% 1.00-1.04; Fig. 5I).

### Subtype-specific immune architectures reveal spatial reorganization of lymphoma microenvironment.

Although these analyses defined distinct cellular compositions associated with hot and cold TME states, they did not resolve how these populations are spatially organized into functional microenvironmental niches. We therefore leveraged cell-cell interaction analyses to reconstruct the spatial architectures that govern TME organization across lymphoma subtypes (**Fig. S8A**). CD4^+^ Tm cells were more frequently localized near B cells in FL1-3A compared with DLBCL (p = 0.03, **Fig. S8B**). Similarly, B cells showed increased proximity to CD8^+^ naïve-like T cells in FL1-3A and GCB DLBCL than in FL3B (p = 0.033). CD8^+^ Tregs were preferentially close to B cells across both FL subtypes and ABC DLBCL relative to GCB DLBCL (p = 0.015, **Fig. S8C**), whereas M1-like macrophages showed increased adjacency to B cells in FL3B (p = 0.027, **Fig. S8D**). NK-B cell spatial distance varied markedly across subtypes, with closest association in FL3B, intermediate in FL1-3A, and the greatest separation in GCB DLBCL (p = 0.04), indicating progressive spatial decoupling of innate and adaptive compartments along disease progression.

Next, we examined spatial co-occurrence involving malignant and GC-derived B-cell subsets. Malignant B cells (CD45^+^CD20^+^BCL2^+^) were more frequently located near CD4^+^ Tn cells in FL1-3A than in GCB DLBCL (p = 0.008; **Fig. S8E**). CD4^+^ Tn cells also showed closer proximity to LZ B cells in FL1-3A and FL3B compared with GCB DLBCL (p < 0.01). Similarly, CD4^+^ Tm cells and mDC were enriched near LZ B cells, with stronger CD4^+^ Tm interactions in FL1-3A and FL3B (p = 0.02 and p = 0.006, respectively), whereas mDC-LZ proximity was higher in GCB DLBCL (p = 0.03; **Fig. S8F**). Among CD8^+^ subsets, Tn cells showed intermediate spatial association with LZ B cells in FL1-3A relative to GCB DLBCL (p = 0.04). CD8^+^ Tregs were consistently enriched near LZ B cells in both FL subtypes (FL1-3A p = 0.04, and FL3B p = 0.04) and additionally showed increased proximity to DZ B cells in FL3B compared with FL1-3A and DLBCL (p = 0.05 and p = 0.03, respectively). In contrast, monocytes displayed stronger spatial association with both LZ and DZ cells in GCB DLBCL than in FL1-3A across multiple spatial scales (p = 0.04 and p = 0.04, respectively), indicating a shift towards myeloid-centered interaction networks in aggressive disease states. In FL3B, monocytes and CD4^+^ Tem were frequently co-localized with PC-like populations (**Fig. S8G**), whereas NK cells were generally spatially segregated from B cell-compartments than in other lymphoma subtypes (**Fig. S8H**). Collectively, these data define subtype-specific immune architectures characterized by progressive remodeling of B-T cell spatial organization. FL1-3A retains tightly structured B-T cell neighborhoods consistent with GC-like organization, whereas FL3B and DLBCL display progressive spatial decoupling of lymphocyte interactions accompanied by increased engagement of myeloid and NK compartments.

### Spatially Resolved Cellular Communities define Tumor Microenvironment States and Prognosis.

**Figure 6:**
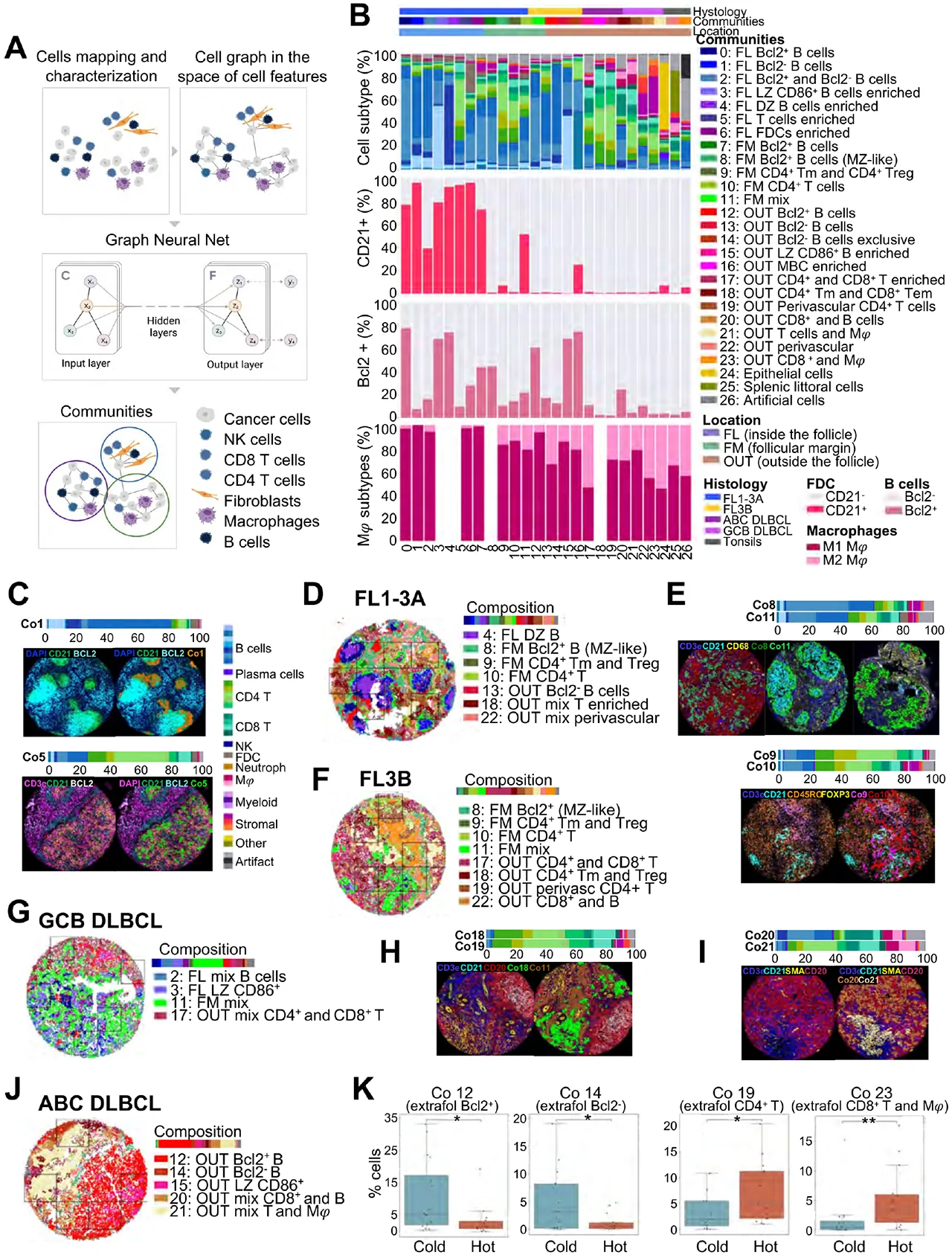
Spatially resolved Cellular Communities define Tumor Microenvironment States. **A.** Schematic overview of spatial cell mapping and graph neural network-based identification of cellular communities from multiplexed CODEX imaging. **B.** Distribution of cell subtypes, CD21 and Bcl2 positivity, and macrophage (M*φ*) subtypes across 27 spatially defined cellular communities. **C.** Representative CODEX images of selected markers within intrafollicular (‘FL’) communities (Co1 and Co5). **D.** Community composition in a representative FL1-2 case. **E.** Representative CODEX images of communities localized to the follicle margin (‘FM’), including Co8 and Co11 (top), Co9 and Co10 (bottom). **F-G.** Community composition in representative FL3B (**F**) and GCB DLBCL (**G**) cases. **H-I.** Representative CODEX images of extrafollicular (‘OUT’) communities, including Co18 and Co19 (**H**), Co20 and Co21 (**I**). **J.** Community composition in a representative ABC DLBCL case. **K.** Quantification of the relative abundance of the indicated cellular communities in TME^hot^ vs. TME^cold^ states. Statistical significance was assessed using the Kruskal-Wallis test followed by Dunn's multiple-comparison test with Benjamini-Hochberg correction. *, P < 0.05; **, P < 0.01.

To move beyond pairwise spatial interactions and capture higher-order tissue organization, we applied a graph neural network-based approach to infer multicellular spatial communities within the lymphoma microenvironment. Using MxIF imaging data, this framework enabled unsupervised identification of spatially coherent cellular communities and quantification of their abundance across samples (Fig. 6A). This analysis revealed 27 distinct communities (Co), defined by cellular composition and expression of CD21, BCL2, and macrophage polarization states (M1/M2-like) (Fig. 6B, **Fig. S9A-C** and **Suppl Table S9**). Seven communities (Co0-6) corresponded to follicular regions characterized by high CD21 expression (labeled as ‘FL’). Within this group, Co1 consisted mainly of BCL2^−^ B cells within GC-like structures, consistent with residual non-neoplastic follicles, whereas Co5 was primarily composed of CD4^+^ and CD8^+^ T cells within follicular regions (Fig. 6C). The follicular communities were significantly more prevalent in FL1-3A compared to other lymphoma subtypes, consistent with progressive loss of GC-like structures during disease evolution (Fig. 6D and **Fig. S9D-F**). Five communities (Co7–11) localized at the follicle margin (labeled as ‘FM’) and were shared across high-risk FL1-3A, FL3B and DLBCL. Among these, Co8 was predominantly composed of BCL2^+^ B cells and was markedly enriched in ABC compared with GCB DLBCL (7.3% vs 1.2%, p = 0.04), representing a major fraction of its tumor microenvironment (Fig. 6E and **Fig. S9G**). Co9 and Co10 were CD4^+^ T cell-enriched communities, with Co9 showing enrichment of Tm and Tregs and higher prevalence in FL1-3A and FL3B compared to ABC and GCB DLBCL (5.7% and 8.7% vs 1.8% and 1.3%; p = 0.04). However, in FL3B these communities exhibited a more diffuse spatial organization than in FL1-3A (Fig. 6E-F and **Fig. S9G-H**), suggesting loss of follicular compartmentalization. Co11 contained mixed B cells, T cells, and macrophages, showing higher abundance in FL1-3A and GCB DLBCL compared to ABC DLBCL (8.1% and 20.9% vs 0.9%; p = 0.02) (Fig. 6G and **Fig. S9G-I**). Intriguingly, Co11 was predominantly located along the inner follicular boundary in low-grade FL, whereas it exhibited a broader, pseudo-perivascular distribution in GCB DLBCL, consistent with progressive architectural disorganization. Twelve communities (Co12–23) were located outside follicular structures (designated as 'OUT') and were mainly observed in high-risk DLBCL. Within this group, Co18 and Co19 were enriched in CD4^+^ Tem and Tregs, and were more frequent in FL1-3A and FL3B compared to DLBCL (p < 0.05 and p = 0.01, respectively; Fig. 6H and **Fig. S9J**). Conversely, Co20 and Co21, composed of mixed T cells and macrophages, were preferentially expanded in ABC DLBCL (p = 0.006 and p = 0.03; respectively; Fig. 6I-J and **Fig. S9K**). The remaining communities (Co24–26) corresponded to epithelial, splenic, or artifact regions and were excluded from downstream analyses.

We next assessed how these spatial communities map onto hot and cold TME states. Consistent with B-cell enrichment in immune-depleted states, Co12 and Co14 - enriched for extrafollicular BCL2^+^ and BCL2^−^ B cells, respectively - were prevalent in cold TMEs (p < 0.05). In contrast, Co19 and Co23 - characterized by extrafollicular CD4^+^ T cells and mixed CD8^+^ T cells with macrophages, respectively - were preferentially enriched in hot TMEs (p < 0.01; Fig. 6K).

Finally, spatial organization of cellular communities was strongly associated with clinical outcome. Higher abundance of Co0 (intrafollicular BCL2^+^ B cells) correlated with inferior EFS (HR 1.12, CI 95% 1.02–1.22, p = 0.011), whereas Co14 (extrafollicular BCL2^−^ B cells) was associated with improved EFS (HR 0.4, CI 95% 0.16–0.97, p = 0.045; **Fig. S9L**). Similarly, Co8 (perifollicular BCL2^+^ B cells) was linked to an increased risk of failing EFS24 (p < 0.05), whereas Co6, Co7, and Co11 were associated with successful achievement of this endpoint (p < 0.05). Importantly, Co4 (intrafollicular DZ B cells) was associated with inferior OS across subtypes (HR 1.27, CI 95% 1.00-1.62, p = 0.049), with a stronger effect in FL (HR 2.82, CI 95% 1.07–7.42, p = 0.035) (**Fig. S9M**). Collectively, these findings establish a hierarchical architecture of lymphoma TME, in which B-cell-rich and DZ-enriched communities organize immune-depleted niches, whereas extrafollicular T-cell and myeloid-dominant communities form spatially coordinated immune-enriched ecosystems that stratify patient outcome.

### Malignant B-cell differentiation state instructs lymphoma microenvironment in vivo.

**Figure 7:**
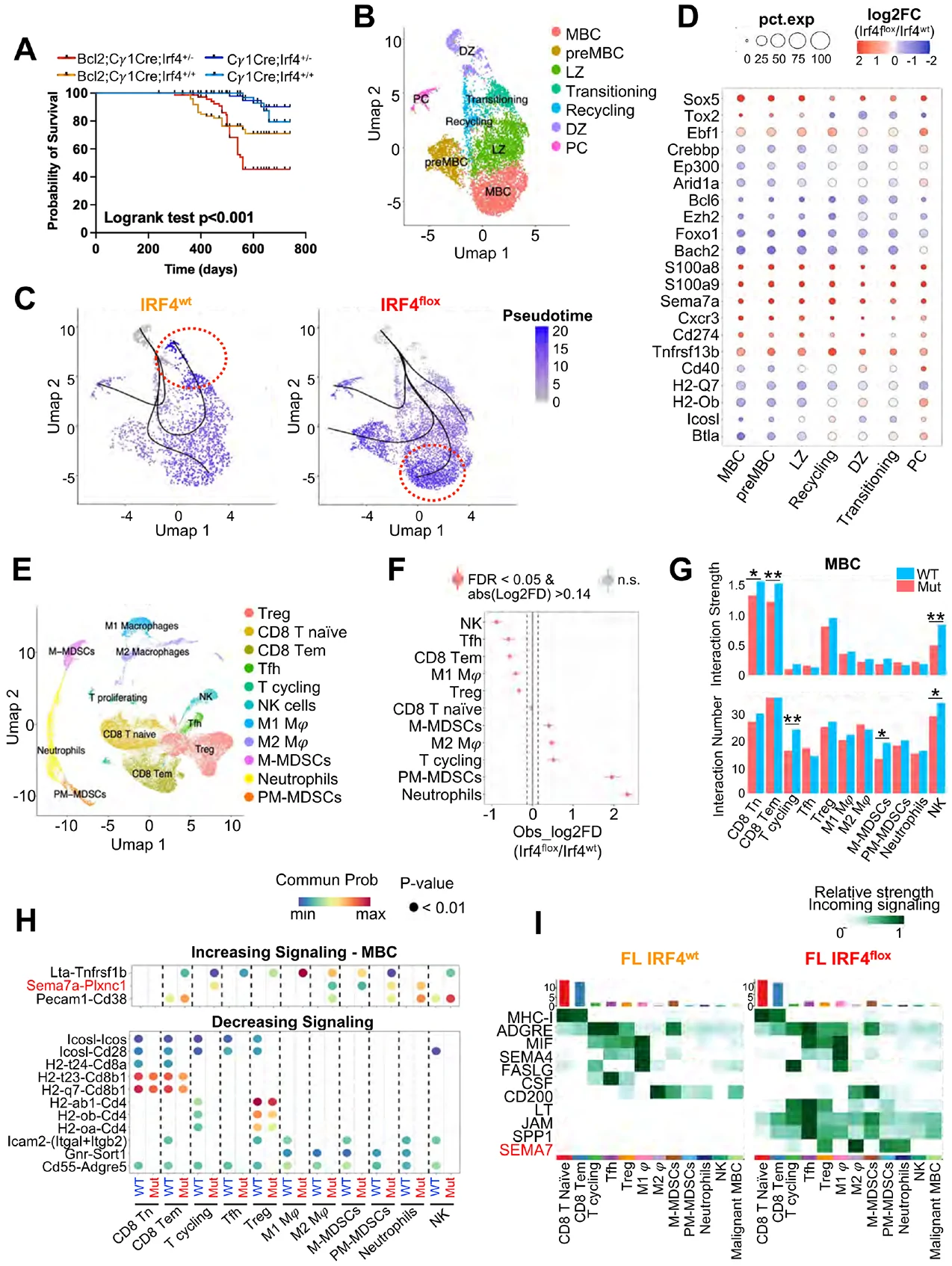
Malignant B-cell differentiation states shape the lymphoma microenvironment *in vivo*. **A.** Kaplan-Meier survival curves of lymphoma-bearing mice and controls with intact or deleted IRF4. **B.** UMAP projections of malignant B cells from mice in (**A**), with clusters color-coded and annotated by cellular phenotype. **C.** Pseudotime trajectory analysis of malignant B cells inferred using Slingshot. **D.** Bubble plot showing differential expressed genes between malignant B-cell clusters in IRF4-deficient vs wild-type (WT) mice. Circle color indicates average expression level; circle size reflects statistical significance. **E.** UMAP projections of immune subsets from mice in (**A**), with clusters color-coded and annotated by cellular phenotype. **F.** Comparison of immune subset composition between IRF4-deficient and WT mice. **G.** Cell-cell interaction strength (top) and number (bottom) between immune clusters and malignant memory B cells (MBCs) in IRF4-deficient (red) and WT (blue) mice. **H.** Ligand-receptor interaction analysis between immune clusters and malignant MBCs; dot color indicates interaction probability, and absence of dot indicates no detectable interaction. **I.** Inferred incoming signaling activity of selected pathways across immune clusters and malignant MBCs in IRF4-deficient (right) and WT (left) mice. Statistical significance was assessed using the Kruskal-Wallis test followed by Dunn's multiple-comparison test with Benjamini-Hochberg correction. *, P < 0.05; **, P < 0.05.

To directly test whether malignant B-cell differentiation state governs immune microenvironment organization, we generated a genetically engineered lymphoma mouse model enabling B-cell-restricted modulation of IRF4 by crossing *Irf4*-floxed mice^21^ with *C γ 1-Cre* and *Bcl2-Ig*^46^ transgenic strains. As a master regulator of PC differentiation and GC fate decisions^21,47,48^, IRF4 provided a tractable mechanism to experimentally manipulate malignant differentiation *in vivo*. We established cohorts of lymphoma-bearing mice with intact and deleted IRF4 (*Bcl2;C γ 1-Cre;Irf4*^+/+^ and *Bcl2;C γ 1-Cre;Irf4*^−/+^) alongside non-malignant GC controls (*C γ 1-Cre;Irf4*^+/+^ and *C γ 1-Cre;Irf4*^−/+^) (n = 25 mice per genotype). Following immunization with sheep red blood cells, mice were monitored longitudinally for up to two years (**Fig. S10A**). Both lymphoma cohorts showed inferior survival compared to controls, with *Bcl2;C γ 1-Cre;Irf4*^−/+^ mice displaying the most aggressive disease course (p < 0.001; Fig. 7A). Consistent with the established role of IRF4 in PC commitment^21,47^, IRF4 loss resulted in marked depletion of PCs (B220^+^CD138^+^, p < 0.0001) and reciprocal expansion of MBC populations (B220^+^CD38^+^FAS^−^CD138^−^IgD^−^, p = 0.020; **Fig. S10B**), confirming effective reprogramming of malignant B-cell fate. To define the consequences of this lineage shift, we performed paired scRNA/BCR-seq on splenic tumors from 4 mice per genotype. UMAP embedding separated B cells from immune compartments, while VDJ clonality enabled identification of malignant B cells for downstream analysis (**Fig. S10C**). Reclustering resolved seven transcriptionally distinct GC-derived^49^ malignant states (Fig. 7B). *Bcl2;C γ 1-Cre;Irf4*^−/+^ tumors were enriched for pre-MBC and MBC-like populations (FDR = 0.001), whereas DZ-like and transitioning states predominated in *Bcl2;C γ 1-Cre;Irf4*^+/+^ lymphomas (FDR = 0.004 and 0.001; **Fig. S10D**). Trajectory inference^50^ confirmed divergent terminal fates, with MBC-like cells representing the dominant terminal state in IRF4-deficient tumors and DZ-like cells defining the terminal state in control lymphomas (Fig. 7C). This shift in malignant cell identity was accompanied by extensive transcriptional reprogramming. IRF4-deficient malignant B cells upregulated genes associated with B-cell differentiation (e.g., *Sox5, Tox2, Ebf1*), while suppressing canonical DZ-associated regulators (e.g., *Bcl6, Ezh2, Foxo1*). Notably, MBC-like tumors also exhibited increased expression of immune suppression molecules (e.g., *S100a8/9, Sema7a, Cd274*), together with reduced expression of antigen presentation machinery (e.g., *H2-q7, H2-Ob*) and co-stimulatory molecules (e.g., *Cd40, Icosl, Btla*; Fig. 7D). Consistent with these changes, GSEA demonstrated activation in NOTCH signaling and suppression of immune response pathways (**Fig. S10E**), indicating acquisition of an immune-evasive transcriptional program.

Strikingly, malignant state reprogramming was accompanied by profound remodeling of the immune microenvironment. IRF4-deficient tumors displayed reduced CD4^+^ T cells abundance (p = 0.011) and expansion of CD8^+^ T cells (p = 0.001), together with near-complete loss of Tfh cells (p < 0.0001). The residual Tfh compartment exhibited increased expression of exhaustion markers (CD4^+^CXCR5^+^PD1^+^TIM3^+^; p = 0.014), while both total and activated Tregs were significantly expanded (CD4^+^CD25^+^PD1^+^; p < 0.0001 and CD4^+^CD25^+^PD1^+^TIM3^+^; p = 0.039; **Fig. S10F-G**). Single-cell profiling of the immune compartment identified 11 immune subsets and revealed enrichment of cycling T cells and suppressive myeloid populations, including neutrophils, PM-MDSCs, M-MDSCs, and M2 macrophages in *Bcl2;C γ 1-Cre;Irf4*^−/+^ mice (Fig. 7E-F and **S10H**). These features closely recapitulated the inflamed yet dysfunctional TME observed in human MBC-like lymphomas. To determine how malignant state reprogramming alters tumor-immune communication, we performed CellChat analysis^44^. IRF4-deficient tumors displayed substantially increased malignant-immune crosstalk (Fig. 7G) characterized by attenuation of MHC-dependent antigen presentation and canonical co-stimulatory signaling, accompanied by enrichment of inhibitory signaling pathways, including SEMA7A-PLXNC1, LTA-TNFRSF1A/B, and PECAM1-CD38/CD177 (Fig. 7H and **S10I**). Among these, SEMA7 signaling emerged as a dominant communication network spanning multiple immune populations (Fig. 7I), identifying a candidate immunoregulatory axis associated with the MBC-like state. By contrast, DZ-like lymphomas showed comparatively limited immune remodeling, primarily mediated by inhibitory CD200-CD200R and GNR-SORT1 signaling, while largely preserving antigen presentation interactions (**Fig. S10L-M**). Although SEMA7 signaling was detectable in both states, its activity was markedly amplified in MBC-like tumors (**Fig. S10N**). Together, these findings establish malignant B-cell differentiation state as a casual determinant of the TME. Experimental enforcement of an MBC-like fate was sufficient to rewire tumor-immune communication, suppress antigen presentation, and drive the formation of a profoundly immunosuppressive microenvironment, demonstrating that immune architecture is actively dictated by malignant lineage state rather than passively acquired during lymphoma evolution, providing direct evidence that differentiation programs within malignant B cells govern the formation of distinct lymphoma microenvironments.

## DISCUSSION

Through integrated bulk and single-cell transcriptomics, spatial proteomics, genomic profiling, and functional analyses of human and murine GC-derived lymphomas, we identified malignant B-cell differentiation state as a fundamental determinant of the lymphoma microenvironment. Across FL and DLBCL, tumors did not segregate according to conventional histologic boundaries but instead aligned along a conserved developmental continuum spanning proliferative DZ-like states with immune-depleted microenvironments and post-GC/MBC-like states with inflamed but profoundly immunosuppressed ecosystems. These findings support a model in which malignant lineage identity actively shapes immune composition, tissue architecture, and mechanisms of immune escape during lymphoma evolution.

A central finding of this study is that the malignant state provides a unifying framework for understanding lymphoma ecosystem diversity. Previous studies have described inflammatory, immune-depleted, or stromal-rich microenvironments as distinct lymphoma states.^15-18,40^ Our analyses suggest that these ecosystems are not discrete entities but are instead organized along a shared developmental trajectory. Immune-depleted TMEs consistently aligned with proliferative DZ-like malignant programs characterized by BCL6/FOXO1 and cell-cycle activity, whereas immune-enriched TMEs were associated with post-GC/MBC-like states marked by IRF4 activation, inflammatory signaling, and activated yet exhausted T-cell programs.^7^. Importantly, these developmental states engaged fundamentally different mechanisms of immune evasion. DZ-like tumors exhibited features of immune exclusion, including impaired antigen-presentation and recurrent alterations affecting HLA genes, whereas MBC-like tumors displayed evidence of chronic immune engagement accompanied by T-cell dysfunction, suppressive myeloid remodeling, and activation of inhibitory signaling networks. Thus, malignant differentiation state appears to determine not only the composition of the immune microenvironment but also the dominant mechanisms though which tumors evade immune control, establishing developmental identity as a core organizing principle of lymphoma immune architecture (**Fig. S11**).

Single-cell trajectory analyses further revealed that FL and DLBCL occupy a shared DZ-to-MBC developmental continuum. Despite substantial clinical and molecular heterogeneity, all major GC-derived lymphoma subtypes originated from a common DZ-like state and diverged toward distinct terminal differentiation programs associated with fundamentally different microenvironments. Indolent FL1-2 largely occupied transitioning and antigen-presenting states, whereas GCB DLBCL remained locked in proliferative DZ-like programs characterized by FOXO1, BCL6, and EZH2 activity. In contrast, ABC DLBCL and a subset of FL3B preferentially converged toward MBC-like states enriched for inflammatory and immunosuppressive signaling. These findings suggest that lymphoma evolution is driven not only by progressive acquisition of genetic alterations but also by directional remodeling of malignant differentiation trajectories that continuously reshape tumor-immune interactions. Within this framework, genetic lesions may influence disease behavior by stabilizing specific developmental states and their associated microenvironmental programs.

This developmental framework also provides biological insight into the longstanding ambiguity surrounding FL3B^51-53^. Rather than representing a uniform clinicopathologic entity, FL3B segregated into at least two biologically distinct states positioned between low-grade FL and DLBCL. One subgroup displayed MBC-like trajectories and inflammatory programs resembling ABC-like biology, whereas another retained LZ-associated and antigen-presentation programs more closely aligned with low-grade FL. The convergence of transcriptional, genomic, and spatial features argues that FL3B represents a transitional evolutionary state along the trajectory of histologic transformation rather than a discrete disease entity. More broadly, these findings suggest that lymphoma progression reflects dynamic remodeling of developmental identity accompanied by progressive rewiring of immune ecosystems.

Spatial profiling further demonstrated that developmental state is encoded not only in transcriptional programs but also in tissue architecture. Low-grade FL retained structured follicular and perifollicular immune niches enriched for coordinated B-T cell interactions, preserving key features of physiologic GC organization. In contrast, DLBCL displayed progressive dissolution of follicular architecture accompanied by expansion of extrafollicular myeloid-rich and immunosuppressive cellular communities. These transitions were particularly evident in FL3B, where redistribution of regulatory T-cell and myeloid populations reflected an intermediate state of architectural collapse. These observations indicate that malignant differentiation programs are closely coupled to higher-order tissue organization and support a model in which lineage state directly influences immune cell recruitment, localization, and niche formation during lymphoma progression.

A major advance of this study is the demonstration that malignant differentiation state is sufficient to drive immune microenvironmental remodeling. By genetically enforcing an MBC-like fate in a murine lymphoma model, we induced a profound shift toward an inflamed yet highly immunosuppressive microenvironment characterized by Tfh exhaustion, Treg expansion, accumulation of suppressive myeloid populations, and broad attenuation of antigen presentation and co-stimulatory signaling. Conversely, DZ-like lymphomas exhibited comparatively limited immune remodeling despite retaining inhibitory signaling networks. These findings establish a direct causal link between malignant lineage state and immune ecosystem architecture and support an emerging paradigm in which developmental cell state functions as a dominant regulator of tumor-host interactions^54^.

Among the mechanisms associated with this state-dependent immune remodeling, SEMA7A emerged as a prominent immunoregulatory axis. Across both human and murine systems, MBC-like lymphomas displayed extensive activation of SEMA7A-mediated communication networks involving T cells, NK cells, and suppressive myeloid populations. This occurred in parallel with attenuation of canonical MHC-dependent and co-stimulatory interactions, suggesting that immune dysfunction in these tumors is driven not only by impaired antigen presentation but also by active reinforcement of inhibitory circuits. Although SEMA7A has been implicated in immune regulation and tumor progression in solid malignancies^55-58^, its role in lymphoma biology has remained largely unexplored. Our findings therefore identify SEMA7A signaling as a previously unrecognized regulator of post-GC lymphoma ecosystems and nominate this pathway as a potential therapeutic vulnerability in immune-inflamed but dysfunctional lymphomas.

These observations have important translational implications. Current immunotherapeutic paradigms often assume that immune-infiltrated tumors are inherently more responsive to checkpoint blockade. Our data challenge this assumption by demonstrating that immune-enriched lymphomas frequently harbor profoundly suppressive and dysfunctional microenvironments. This may partially explain the modest efficacy of checkpoint inhibition observed in unselected FL and DLBCL populations^59,60^. Stratification based on malignant differentiation state and spatial immune architecture may therefore provide a more biologically informed framework for therapeutic selection than histology alone. In particular, MBC-like hot tumors may require combinatorial approaches targeting suppressive myeloid circuits, SEMA7A signaling, or antigen-presentation defects in addition to checkpoint inhibition, whereas DZ-like cold tumors may benefit from strategies aimed at restoring immunogenicity and enhancing immune recognition. More broadly, our findings raise the possibility that therapeutic manipulation of malignant cell state could be leveraged to reprogram the lymphoma microenvironment and restore antitumor immunity.

Several limitations warrant consideration. Although this study represents one of the largest integrated multiomic analyses of GC-derived lymphomas to date, some subgroup analyses, particularly within FL3B and unclassified DLBCL, remain limited by sample size. In addition, while our murine model establishes causality between malignant differentiation state and immune remodeling, it does not fully recapitulate the genetic complexity of human lymphoma evolution. Future studies integrating longitudinal sampling, lineage tracing, and therapeutic perturbation will be important to define the temporal dynamics and reversibility of lineage-state-dependent immune remodeling.

In summary, we define a unifying developmental model for GC-derived lymphoma evolution in which malignant B-cell differentiation state functions as a master regulator of immune ecosystem organization. Across FL and DLBCL, tumors occupy a shared developmental continuum linking DZ-like proliferative states with immune exclusion, and post-GC/MBC-like states with inflamed but profoundly dysfunctional immune environments. By integrating human multiomic profiling with mechanistic genetic perturbation *in vivo*, we demonstrate that malignant lineage state is not merely associated with immune remodeling but is a causal determinant of immune architecture, spatial organization, and immune escape. Together, these findings establish malignant cell identity as a fundamental organizing principle of tumor-immune co-evolution and identify lineage-directed reprogramming as a potential therapeutic strategy to restore antitumor immunity in lymphoma.

## METHODS

### Patients

Patients with newly diagnosed FL1-3A (n = 74)^18^, FL3B (n = 10) and DLBCL (n = 304)^20^ were prospectively enrolled in the Lymphoma SPORE Molecular Epidemiology Resource (MER) cohort at the Mayo Clinic and University of Iowa and included in this study, with clinical characteristics detailed in **Supplementary Tables S3–S5**. An equal number of male and female subjects were included to avoid sex bias. Patients were older than 18 year, white and selected based on institutional availability. The study was not blind or randomized. Histologic diagnosis was performed according to the World Health Organization criteria by an expert pathologist at each participating center. The clinical stage of the disease was determined based on the medical history and physical examination; complete blood count; serum biochemical profile; CT scan of the chest, abdomen, and pelvis; PET scan with FDG whenever available; and bone marrow core biopsy of the iliac crest as per standard institutional guidelines. Patients were classified into TME^hot^ and TME^cold^ groups based on unbiased gene expression deconvolution, as specified below. All patients were systematically followed for disease progression/relapse, re-treatment, and death; all events were validated against medical records. The proposed study as powered to detect biologically meaningful differences in transcriptional profiles and immune microenvironment features according to TME status. The patients signed written informed consent to participate in the MER cohort study, and this study was reviewed and approved by the local institutional ethics committees. The study was conducted according to the Declaration of Helsinki.

### Mouse Models

The Institutional Animal Care and Use Committee (IAUCUC) of Mayo Clinic approved all mouse procedures (Protocol A00006450-22; approved 03/11/2022). Conditional *irf4*^fl/fl^ knock-out mice^21^ (RRID:IMSR_JAX:009380) and *Bcl2-Ig* transgenic mice^34,46^ (RRID:IMSR_JAX:002321) were obtained from the laboratory of Riccardo Dalla-Favera. To generate lymphoma-bearing and germinal center models with intact or deleted IRF4 expression, *irf4*^fl/fl^ were crossed with *Bcl2-Ig* and C *γ* 1-cre mice (RRID:IMSR_JAX:010612).

### TME Deconvolution for FL and DLBCL Tissues

Bulk RNA-seq data from newly diagnosed FL and DLBCL samples were processed using previously published methods^18,20^. Gene expression counts were scaled using library depth, normalized using trimmed means of M-values method, and log-transformed. When multiple transcripts mapped to the same gene, the transcript with the highest expression level was retained for downstream analysis. The resulting cell-type gene expression matrix was loaded into the ConsensusTME package^61^ to estimate the relative abundance of immune cell populations within each sample. The B-cell signature within the reference gene sets was further refined by retaining only genes whose expression correlated with that of normal B cells, as previously described^61^. Processed gene expression profiles were scored against the refined cell-type signatures using singscore method^62^ to generate sample-level enrichment scores representing the relative abundance of immune cell populations. For downstream analyses, abundance score for each immune cell type were standardized using z-score transformation across samples. Tumor microenvironment (TME) states were classified as TME^hot^ or TME^cold^ based on the cumulative z-score of T-cell and macrophage signatures, with zero as the threshold for classification. In parallel, a probabilistic TME score was computed as the aggregate of inferred immune cell proportions per samples, capturing the degreed of immune enrichment along a continuum.

### Bulk RNA-seq Analysis

RNA-seq data from sorted CD19^+^ B cells from newly diagnosed FL samples and bulk RNA-seq data from newly diagnosed DLBCL samples were processed using previously published pipelines. FL samples were processed using the MAPR-Seq^63^ pipeline configured with hg38 reference and the Ensemble v78 transcriptome annotation. DLBCL samples were aligned to the hg38 reference genome using the STAR aligner^64^ and transcript abundance was quantified using Salmon^65^ (RRID:SCR_004463). For downstream analyses, samples were stratified into TME^hot^ vs TME^cold^ groups as described above. Gene-level count data were extracted and input into edgeR software^66^ (RRID:SCR_012802) for assessing differential expression between the two groups using the quasi-likelihood method. Genes with a differential expression FDR q value of < 0.05 were considered as statistically significant. Gene expression was visualized using log2(TPM + 1) values. For pathway enrichment analysis, differential expression results were imported into the R environment, and a ranked score was calculated for each gene using the formula: −log10(differential expression p value) × log2(FC). Gene symbols and their corresponding rank scores were used as input for gene set enrichment analysis using fgsea R package^67^. Enrichment was assessed for gene sets form HALLMARK, REACTOME (RRID:SCR_003485), and Gene Ontology Biological Process (C5:GOPB) collections. Gene sets with an FDR q value of < 0.05 were considered to be significantly enriched and reported.

### Hypergeometric Analysis of Enriched Gene Sets

The gene sets with a statistically significant enrichment in a group comparison were reassessed using stringent hypergeometric analyses^68^. For this, the differentially expressed genes in the corresponding comparison were filtered to retain genes with an adjusted P value of < 0.05. Next, the number of upregulated and downregulated genes was separately enumerated for each candidate gene set. Separate standard hypergeometric tests were conducted to measure the significance of upregulation or downregulation of the gene set when using significantly differentially expressed genes. The resulting upregulation and downregulation P values for each candidate gene set were adjusted using the Benjamini–Hochberg method. The adjusted P values were log-transformed and summarized.

### Whole-Exome Sequencing

Whole exome sequencing (WES) was performed on tumor–normal pairs from 113 newly diagnosed patients with FL and 267 patients with DLBCL, as previously described^18,20^. FL and DLBCL samples were processed and analyzed independently. For FL samples, matched tumor and germline DNA libraries were prepared using the Agilent SureSelect XT Human All Exon v5 + UTR capture kit and sequenced on an Illumina NovaSeq 6000 platform with 150bp paired-end reads. For DLBCL samples, libraries were prepared using the Agilent SureSelect Human All Exon v6 and sequenced on an Illumina HiSeq2500, 100bp paired-end reads. Sequencing reads from both sets were adapter-trimmed and aligned to the human reference genome (GRCh38/hg38) using BWA-MEM^69^. Base quality score recalibration and duplicate reads removal were performed using standard best-practice workflows. Tumor-normal kinship was confirmed for each pair prior to variant calling. Somatic variants in FL were identified using MuTect (RRID:SCR_000559), JointSNVMix (RRID:SCR_006804), and SomaticSniper^70-72^. For DLBCL samples, somatic variants were called using TNhaplotyper (Sentieon implementation of MuTect2[(RRID:SCR_026692)])^73^. Variants with low variant allele frequency (VAF), low quality scores, or those commonly observed in population SNP databases were excluded from downstream analysis. Enrichment of genetic alterations between groups was assessed using Fisher’s exact tests. Mutation data were analyzed and visualized using the R packages maftools^74^, forestplot^75^, and ComplexHeatmap^76^.

### Transcriptomic and Genomic Classification

Lymphoma genetic subtype classifications were generated using LymphGen^39^, LME^15^ and EcoTyper^40^ as previously described^20^. The DLBClass algorithm^38^ was applied to a subset of DLBCL samples for which *BCL2, BCL6*, and *MYC* translocation status, mutation profiles, and CNV data were available. Refined cell-of-origin classification incorporating the dark zone (DZ) signature and microenvironment archetype profiling were derived from gene expression count data using the R packages gneSegCOO^77^ v1.0 and LymphoMapR^16^, respectively. The distribution of genetic and transcriptomic classes among TME^hot^ and TME^cold^ conditions was visualized using the ggplot2 R package (RRID:SCR_014601).

### CODEX Analysis

#### Cohort description

To investigate the spatial organization across the lymphoma spectrum, we performed multiplex immunofluorescence (MxIF) imaging using the PhenoCycler-Fusion platform on tissue microarray (TMA) constructed from 42 patients, each represented in technical duplicates, yielding 98 tissue cores in total. The cohort included 20 cases of FL1-3A, 9 cases of FL3B and 13 cases of DLBCL (GCB, n = 7; ABC, n = 6). Control tissues consisted of tonsil and spleen samples from 5 healthy donors, as well as liver tissue and peripheral blood mononuclear cells (PBMCs) from two additional healthy individuals. During the slide preparation, 10 cores were exhausted, leaving 88 cores available for analysis. The quality control of tissue integrity, signal specificity and staining was performed by a board-certified pathologist. An additional 9 cores were excluded due to technical artifacts, including tissue damage (e.g., crushing), out-of-focus imaging, or artifact coverage exceeding 80% of the tissue area. The final dataset comprised 79 evaluable cores, representing 30 lymphoma patients and control tissues. Specifically, this included FL1-3A (n = 19 patients, 34 cores), FL3B (n = 8 patients, 13 cores), and DLBCL (n = 12 patients, 22 cores [GCB, n = 6 patients and 11 cores; ABC, n = 6 patients and 11 cores]), as well as control samples from 4 tonsils, 4 spleens, and 2 lives tissues. The block was stained with a panel of 51 antibodies (**Supplementary Tables S8**), of which 48 demonstrated specific and clear signals. Three markers (CD31, GATA3, and Tbet) did not pass the pathology quality control stage and were excluded from the analysis.

### MxIF image analysis: Cellular segmentation

We performed cell instance segmentation using a U-Net + + ^78^ convolutional neural network (CNN). The model’s input consisted of a composition of DAPI and NaK-ATPase (as nuclear and membrane markers, respectively), to generate individual cell segments. To ensure comprehensive cell identification for the majority of cells, a slight expansion around the nuclear region was applied for cells with less distinct membrane staining but clear nuclear presence. Segmentation quality was rigorously assessed through visual inspection by pathologists proficient in MxIF analysis. Pathologist’ review confirmed an accuracy exceeding 90% across all samples. To ensure the biological relevance of the detected cells, we implemented size-based filtering to exclude spurious large segments often resulting from staining artifacts, out-of-focus areas, or other artificial anomalies.

#### MxIF image analysis: Generation of specific compartmental signal masks

Signal masks for specific histological compartments were generated using intensity-based thresholding of markers followed by the exclusion of imaging artifacts. Zones with Macrophages and vessels were identified using CD68 and CD34 thresholds, respectively. Stromal zone masks were defined by SMA expression, with CD34 vessel masks subtracted to ensure compartmental specificity. Follicular areas were delineated via CD21 thresholding. To ensure accuracy, all masks underwent quality control. Manual refinement was performed if the automated mask resulted in a > 20% loss of the true signal area or > 20% of the detected area consisted of non-target tissue or artifacts.

### MxIF image analysis: Cell phenotyping

Cell phenotyping was performed based on the distribution of marker signals within the cell contour, using metrics like relative per-cell signal area, defined as the ratio of thresholded marker-positive pixels to the total cell segment area. The entire cell population was clustered using the Leiden algorithm^79^ on the k-nearest neighbor (KNN) graph (number of neighbors = 15). Cell types were assigned to each cluster based on phenotype-defining marker expression. Statistical significance was evaluated using a two-tailed one-sample Wilcoxon signed-rank test. Following this automated annotation, cell type assignments were manually reviewed by a pathologist and corrected as necessary. Additionally, a “gating”-like quality control step was implemented to ensure phenotypic fidelity by comparing marker expression levels against established thresholds, which were determined either through manual image inspection or inferred from cluster-specific signal distributions, thereby excluding cells with inconsistent marker profiles from the final classification. Furthermore, the “gating” strategy was used for defining the positivity of specific markers for statistical analysis. All operations were performed in Python (v3.11) with scanpy framework (v.1.9.8)^80^.

For cell type identification and quantification, cells were segmented using a convolutional neural network and annotated based on the dominant marker expression (Fig. 5A) in 14 major clusters: B cells (CD45^+^CD20^+^), T cells (CD45^+^CD3^+^), macrophages (CD68^+^), monocytes (CD14^+^), natural killers (NK, CD56^+^), plasma cells (CD20^−^CD38^+^), neutrophils (CD66^+^MPO^+^), follicular dendritic cells (FDC, CD20^−^CD21^+^), other dendritic cells (CD11c^+^), lymphatic endothelial cells (podoplanin^+^), endothelial cells (CD34^+^), epithelial cells (Pan^−^CK^+^), splenic littoral cells (CD8^+^CD3^−^) and stromal cells (SMA^+^). We further distinguished B cells in light zone (LZ, CD86^+^ and/or CD83^+^) and dark zone (DZ, CXCR4^+^) subsets and characterized them based on BCL2 expression as normal (CD45^+^CD20^+^BCL2^−^) and malignant (CD45^+^CD20^+^BCL2^+^). The T cells were divided into CD4^+^ and CD8^+^ T cells and each differentiated in regulatory (Treg, FOXP3^+^), memory (Tm, CD45RO^+^), effector memory (Tem, CXCR3^+^) and naïve (Tn, TCF1^+^) phenotypes. Macrophages were classified in M2 and M1 subsets based on the presence or absence of CD163. Additionally, monocyte-derived dendritic cells (mDC) were identified based on the co-expression of CD11c and CD14 within the dendritic cells.

#### MxIF image analysis: Spatial community analysis

Spatial community analysis was adapted from Sarachakov et al^81^ and performed using cells centroid coordinates in conjunction with assigned and cellular phenotypes. Masks for vessels, stromal cells, macrophages and follicles were incorporated in addition to spatial coordinates and cell types. These masks provided supplementary information on the spatial proximity of each cell to a given structural compartment. Then additional spatial features for each cell were computed: distance from a cell to the nearest follicle mask and percentages of other structural masks in the radius of 33 *μ*m. For a subset of B cells, an additional feature of BCL-2 marker positivity was used. Subsequently, a graph represented by an adjacency matrix was constructed using SciPy (v1.15.2) version^82^ (RRID:SCR_008058) of Delaunay triangulation method, incorporating each cell’s centroid coordinates, phenotype and the aforementioned spatial proximity features. Additionally, the edges with length exceeding 152 *μ*m were removed. This graph served as input for an Adversarially Regularized Variational Graph Auto-Encoder (ARGVA)^83^ to obtain a hidden 32-dimensional representation of each cell’s phenotype and spatial characteristics. The auto-encoder was trained for 49 epochs. The model was trained jointly for 49 epochs using the Adam optimizer (learning rate 0.005 for the encoder, 0.001 for the discriminator). Training was performed on the full graph (batch size 1), with mini-batches consisting of sampled nodes (mini batch size was 25000, total number of nodes was 804464). The PyTorch Geometric (v.2.6.1)^84^ version of ARGVA was used. The embeddings generated by the auto-encoder were utilized to perform clustering using the Leiden algorithm^79^ with the resolution 1, resulting in 30 clusters. Evaluation of the resulting spatial clusters involved assessment of cell type composition, marker expression profiles, and their spatial positioning relative to the follicle (inside, outside, or at the border). Notably, 3 specific clusters (epithelial cell, splenic littoral cell, and artificial cell clusters) were defined by their unique cellular characteristics rather than their spatial relationship to follicular structures. **Supplementary Table S9** provides a comprehensive description of the 27 community clusters, including relative positioning to the follicle.

### Co-occurrence calculations

To quantify the spatial organization and interactions between cell types within tissue samples, we calculated a co-occurrence score. This interpretable metric assesses the spatial proximity of two cell types by computing the likelihood ratio of observing them together at a given distance versus observing the target cell type independently. Co-occurrence metrics were calculated using Python (v3.11) with the Squidpy (v1.6.2) framework^85^. For each sample, we determined the co-occurrence between B cells and CD4+, CD8+, and CD68 + cell subtypes across distances ranging from 10 μm to 100 μm. This same approach was then applied to analyze the co-occurrence between B cell subpopulations and the aforementioned CD4^+^, CD8^+^, and CD68^+^ cell subtypes. This methodology enabled the identification of spatial associations and potential differences in cell type interactions across various groups of interest.

### Statistical analysis

Statistical analyses were performed to compare metrics across patients, employing two distinct grouping strategies for diagnostic groups: (1) combining two DLBCL subtypes into a single group, and (2) treating them as separate entities. Further investigations included the division of patients by hot and cold TME samples, different FLIPI and IPI groups, and Event-Free Survival (Achieved/Failed) within 24 months. For patients with multiple samples present in analysis, the mean of each metric across samples was used to represent the patient. All tests were performed in Python (v3.11), using the scikit-posthocs (v.0.10.0) package^86^. Group-wise comparisons across the experimental groups were performed using the Kruskal–Wallis H test, followed by Dunn's post hoc test with Benjamini–Hochberg correction for multiple comparisons. Statistical analyses were conducted in R (RRID:SCR_001905). The assumptions of the Kruskal–Wallis test were assessed prior to analysis. For binary classified samples (e.g., hot vs cold TME, EFS24 achieved vs failed), a two-sided Mann-Whitney U-test was performed followed by Benjamini-Hochberg correction for multiple comparisons. Prior to analysis, the assumptions of the Mann–Whitney U test were evaluated, including random sampling, independence of observations between groups, an ordinal or continuous outcome variable, and similarly shaped distributions across groups. Statistical significance was defined as a two-sided *P* < 0.05. Analyses were performed independently for each diagnostic grouping strategy. Outliers were identified and removed based on visual assessment of signal quality or if less than 10 cells of cell type are present in a sample (only for marker positivity tests).

Analyzed metrics included:

- Cell group proportions - percent of target cell group.
  - Base cell types proportion among all cells in sample
  - B cell subtypes proportion among all B cells in sample
  - CD4 + T cell subtypes proportion among all CD4 + T cells in sample
  - CD8 + T cell subtypes proportion among all CD8 + T cells in sample
  - CD68 + cell subtypes proportion among all CD68 + macrophages in sample
  - Communities proportion among all cells in sample
- Marker-positive cell percentage - percent of positive by certain marker (marker relative per-cell signal area is greater than set threshold) cells.
  - HLA-A, HLA-DR, PD-L1, Ki67 positivity among B cell subtypes
  - CD44, IFNG, Granzyme B, IDO1, VISTA, PD-1, LAG3, TOX positivity among T cell subtypes
  - HLA-A, HLA-DR, PD-L1, Ki67 positivity among B cell-enriched communities
  - CD44, IFNG, Granzyme B, IDO1, VISTA, PD-1, LAG3, TOX positivity among T cell-enriched communities
- Normalized cell density - ratio of cell group percentage in sample and sample area.
  - Normalized cell densities of base cell types
  - Normalized cell densities of subtypes (for B cells, T cells and macrophages)
- Co-occurrence (on distance from 10 μm to 100 μm)
  - Co-occurrence of T cell subtypes and B cells
  - Co-occurrence of macrophages subtypes and B cells
  - Co-occurrence of T cell subtypes and B cells subtypes
  - Co-occurrence of macrophages subtypes and B cells subtypes

### Survival analysis

Overall Survival (OS) and Event-Free Survival (EFS) were analyzed using univariate Cox proportional hazards regression models to evaluate the association between individual covariates and clinical outcome. Survival distributions were visualized using Kaplan–Meier estimates, with patients stratified into groups based on the mean value of the covariate of interest unless otherwise specified. In addition, model-based predicted survival curves derived from Cox proportional hazards regression were generated to illustrate survival probabilities across representative values or proportions of selected covariates. To account for the distinct biological and clinical characteristics of the diseases, FL and DLBCL cohorts were analyzed separately unless otherwise specified. Survival analyses were performed in Python (v3.11) using the lifelines package (v0.28.0)^87^. The proportional hazards assumption was assessed prior to model interpretation. Statistical significance was defined as a two-sided *P* < 0.05.

## SINGLE-CELL ANALYSIS

Fresh-frozen lymph nodes from 21 newly diagnosed patients with FL1-3A^7^ and 8 FL3B were used to scRNA-seq and BCR-seq using the 10X Genomics Chromium platform. Fresh-frozen lymph nodes from 3 FL3B and 13 DLBCL (ABC n = 3; GCB N = 8, Unclassified n = 2) newly diagnosed patients were used for scRNA-seq. Additionally, fresh-frozen lymph nodes from 4 healthy control were used for both scRNAseq coupled with scBCR-seq as well as scRNA-seq only. Briefly, the whole live cells were counted and measured for viability using the Vi-Cell XR Cell Viability Analyzer (Beckman Coulter, Indianapolis, IN). With a desired capture target of 10,000 cells, samples were prepared according to the instructions for the Chromium Next GEM Single Cell 5’ Reagent Kits v2 (10x Genomics, Pleasanton, CA). For cDNA generation, the resulting GEMs were collected and proceeded to reverse transcription, GEM dissolution, and cDNA clean-up. The resulting cDNA, a pool of uniquely barcoded molecules, was used to generate 5’ gene expression libraries. Enriched B-cell receptor (BCR) libraries were also prepared using the Chromium Single Cell V(D)J Amplification Kit (10x Genomics). During library construction, standard Illumina sequencing primers and a unique i7 Sample index (Dual Index TT Set A; 10x Genomics) were added to each cDNA pool (gene expression and BCR). All cDNA pools and final libraries were measured using Qubit High Sensitivity assays (ThermoFisher Scientific, Waltham, MA) and Agilent Bioanalyzer High Sensitivity chips (Santa Clara, CA). Gene expression libraries were sequenced at a minimum of 50,000 read pairs per cell and BCR libraries at a minimum of 10,000 read pairs per cell. Both library types were pooled and loaded across multiple lanes of a NovaSeq S4 flow cell, following Illumina’s standard protocol for the NovaSeq 6000 (San Diego, CA) and using the NovaSeq XP 4-Lane kit for individual lane loading. The flow cells were sequenced as 100 X 2 paired end reads using NovaSeq Control Software v1.8.0. Base-calling was performed using Illumina’s RTA version 3.4.4.

### Sample Integration into a Unified Single-Cell Object

#### 1.1 Quality Control

Quality control was performed in two stages to remove technical artifacts, low-quality cells, and doublets prior to data integration.

Stage 1. Sample-level adaptive filtering using an EM-based model. Each sample was processed individually to accommodate variation in sequencing depth and cell quality. Raw expression matrices were loaded and subjected to initial filtering to remove empty droplets (< 10 UMIs) and cells with very low transcript complexity (< 200 detected genes). To more accurately distinguish viable cells from technical noise, the log-transformed UMI distribution of each sample was modeled using a three-component Gaussian mixture (empty droplets, low-quality cells, and viable cells). Model parameters were estimated with an Expectation–Maximization (EM) algorithm, enabling sample-specific adaptive classification. Only barcodes assigned to the viable-cell component were retained. Following EM-based filtering, potential doublets were identified and removed using Scrublet^88^. This stage yielded a set of per-sample filtered objects with major technical artifacts removed while preserving sample-specific characteristics.

Stage 2. Cohort-level filtering prior to integration. All samples retained after Stage 1 were merged into a single AnnData object and subjected to unified QC criteria to ensure dataset consistency before integration. Cells were required to have 500–10,000 UMIs, a range chosen to exclude remaining low-RNA-content cells as well as potential high-UMI doublets not detected at the sample level. Cells with > 10% mitochondrial transcripts were removed to eliminate stressed or apoptotic cells.

#### 1.2 Harmonization of Single-Cell Transcriptome Profiles in Lymphomas using Conditional Variational Autoencoder (TRVAE)

Integration of samples across lymphoma subtypes was performed with the scArches^89^ framework, using a conditional variational autoencoder (TRVAE) to account for technical variation between cohorts. The FL1–2 samples, representing the largest and most homogeneous group, were used to train the reference TRVAE model. Training produced a 10-dimensional latent space that captures shared biological structure while reducing sample-specific technical effects. The remaining datasets (FL3B, GCB DLBCL, ABC DLBCL, Unclassified DLBCL and normal controls) were subsequently mapped onto this reference using the architectural-surgery procedure implemented in scArches. This allowed new datasets to be incorporated into the latent space without retraining the entire model. After all datasets were mapped, the joint latent representation was used to construct a k-nearest neighbor (KNN) graph followed by Leiden clustering. The integrated structure of the dataset was visualized using UMAP.

#### 1.3 Cell type annotation

The annotation procedure was performed in two stages: an initial broad assignment of major immune lineages followed by a more detailed characterization of B-cell states.

### Broad annotation of all cells

After integration and clustering, the Leiden clusters were assigned to major immune compartments. Cluster identities were determined manually by examining the expression of canonical marker genes on UMAP embeddings (the full list is shown in **Supplementary Table S6**). This step provided a coarse annotation framework, allowing the principal immune populations to be separated for downstream analyses.

### Detailed annotation of B-cell populations

Clusters were annotated manually based on the expression of canonical B-cell marker genes. Additional refinement was guided by differential gene ranking between clusters, enabling the definition of GCB–related differentiation states.

### V(D)J Data Integration

BCR sequencing data were integrated with single-cell gene-expression profiles to link transcriptional states with immune-repertoire information. Clonal architecture was reconstructed for each sample to identify the dominant expanded clone, representing the putative malignant population.

### Data processing and quality control

V(D)J data were imported from the filtered_contig_annotations.csv files for each sample and processed using the scirpy framework^90^. A MuData container was used to store gene-expression and repertoire modalities in a unified structure. Quality control of BCR chains was performed with scirpy functions, and only cells with productive paired heavy and light chains were retained for clonal analysis, ensuring that assignments were based on high-confidence receptor information.

### Clone definition and tumor-clone identification

For each B cell, the amino-acid sequences of the heavy- and light-chain VDJ regions were extracted, and clonotypes were defined based on identical VDJ pairing. The abundance of each clonotype was quantified on a per-sample basis. Clones containing more than 30 cells were considered expanded, and the largest expanded clone within each sample was annotated as the tumor clone, reflecting the principle that lymphoma originates from a single dominant malignant precursor.

### Annotation of tumor and non-tumor cells

Clonal information was added to the metadata of the main AnnData object. Each cell was classified as either 1) tumor-belonging to the dominant expanded clone of its sample; 2) other-B cells belonging to non-dominant (presumably non-malignant) clones; or 3) no VDJ-cells lacking reliable BCR information (e.g., T cells, myeloid cells, or B cells with insufficient V(D)J coverage).

### CNA-based identification of malignant B cells using CNVinfer

InferCNVpy^91^ (RRID:SCR_021140) was used to distinguish malignant from non-malignant B cells in samples lacking BCR information, allowing tumor-cell identification across all DLBCL subtypes (GCB, ABC, and Unclassified). For each subtype, B cells from the tumor cohort were analyzed together with Normal sorted GC B cells, which served as the healthy reference population. The reference group was randomly down sampled to match the number of tumor B cells in each cohort to ensure balanced comparisons.

Subtype-specific gene sets for CNA inference were defined according to genes most frequently altered by copy-number variation in DLBCL. For ABC and Unclassified DLBCL, gene lists were taken from Chapuy et al.^13^, while for GCB DLBCL the gene panel was derived from Reddy et al.^92^, including *BCL2, CREBBP, TNFRSF14, CARD11, GNA13, SOCS1, SGK1, EZH2, TP53, IRF8, PIM1, ARID1A, NOTCH2*, and *B2M*. CNA profiles were inferred using the infercnvpy implementation by comparing each tumour-cohort cell to the mean expression profile of the Normal sorted GC B-cell reference. A cnv_score was computed for every cell, quantifying its degree of inferred genomic instability. For each subtype, a cnv_score threshold was selected so that misclassification of healthy reference cells did not exceed 5%. Cells above this threshold were labelled as malignant, and those below as non-malignant. Cells above this threshold were classified as malignant. Key results are summarized in the table below.

**Table:** 

DLBCL Subtype	Optimal cnv_score Threshold	Identified Tumor Cells	Proportion of Tumor Cells in Cohort (%)
GCB DLBCL	0.23	4,749	42.50%
ABC DLBCL	0.008	2,302	56.00%
Unclassified DLBCL	0.006	1,618	87.60%

### Mutational Profiling Based on Copy-Number Variation

Copy-number variation (CNV) profiles were inferred using infercnvpy^91^. Normal sorted GCB cells were used as the reference population, and tumor cells from each subgroup served as the test set. CNV inference was performed using a sliding window of 50 genes with a 25-gene step, providing high spatial resolution while maintaining manageable noise levels. Prior to CNV calling, expression values were smoothed using a 3-window moving average.

Copy-number gains and losses were defined based on signal amplitude thresholds of ≥ 0.30 and ≤ −0.30, respectively. Adjacent windows showing alterations in the same direction were merged into continuous segments when the combined length was ≥ 3 bins. CNV events were considered significant if they occurred in ≥ 1% of tumor cells. After alignment to chromosome boundaries (SNAP = 1–2) (RRID:SCR_007936) and removal of redundant overlapping regions, a single peak with the highest prevalence was retained for each locus.

### FL3B Subgroup Stratification by Cell Type Proportions

FL3B sample stratification was performed by computing the relative proportions of all cell types identified in step 1.3 for each sample and applying k-means clustering (k = 3) to the resulting sample-by–cell-type matrix. Two clusters containing 4 and 6 samples were identified as distinct FL3B subgroups, whereas one sample formed a separate cluster and was considered an outlier.

### IG-tree analysis

#### BCR repertoire processing and clonal analysis

We analyzed BCR repertoires in two follicular lymphoma groups: classical FL1–2 and FL3B. For each group, 10x Genomics scRNA-seq/VDJ libraries were used, including amino acid and nucleotide sequences of heavy and light immunoglobulin chains together with the 10x annotation tables. All datasets were processed with a single analysis pipeline. References for IGHV, IGHD, IGHJ and IGHC were obtained from IMGT/GENE-DB (RRID:SCR_006964).

FASTA headers were reformatted to be compatible with Ig BLAST/Change-O^93^, and duplicate C-gene entries with identical sequences were removed. For each gene set (V, D, J, C), nucleotide BLAST databases were built using the command makeblastdb -dbtype nucl -parse__seqids. V, D and J gene usage and core immunoglobulin features were annotated with IgBLAST (RRID:SCR_002873) via the Change-O wrapper AssignGenes.py igblast (with --organism human, --loci ig, BLAST output format). The resulting files were converted to AIRR format using MakeDb.py igblast, providing the source FASTA, IMGT V/D/J references and the 10x annotation table (--10x) to merge BCR information with cell metadata; the --extended option was used to retain additional fields. AIRR tables were then imported into R for downstream analysis. BCR-containing cells were mapped back to the Seurat object, retaining only cells corresponding to the states of interest and assigning them to FL1–2 or FL3B. Cell identifiers were harmonized between the BCR tables and the Seurat object.

Quality control of BCR calls was performed in several steps. Cells with more than one heavy (IGH) chain were excluded, as were cells lacking an IGH chain (i.e. with only IGK/IGL). Only productive rearrangements were kept. All subsequent analyses were restricted to the heavy-chain (IGH) sequences. To define an appropriate distance threshold for clone clustering, we examined the distribution of nearest-neighbour (Hamming) distances using distToNearest^94^. A single global threshold of 0.2 was then applied to all samples to ensure consistency between FL1–2 and FL3B.

Clones were defined with function hierarchicalClones (parameters: cell_id = unique cell identifier, threshold = 0.2, only_heavy = TRUE), without merging clones across samples. Condition and sample identifiers were added to the clone summary. Germline IGH sequences were reconstructed using IMGT reference data (readIMGT) and createGermlines.

Phylogenetic trees were inferred using getTrees from the dowser package and visualised with plotTrees (ggtree/ggplot2), displaying isotypes at the tips (tips = "Isotype") and using a branch scale of 0.01.

To examine whether a particular immunoglobulin isotype was preferentially used by the dominant tumor clone, we focused on the most expanded clone in each sample and recorded the isotype most frequently represented within that clone. These dominant-clone isotypes were then compared between FL1–2 and FL3B. Group differences were assessed using two-sided Mann–Whitney U-tests with Benjamini–Hochberg correction.

### Inferring B-Cell Fate Trajectories and Driver Genes via RNA Velocity and CellRank

CellRank^95^ analysis (RRID:SCR_022827) was performed on malignant B cells from follicular lymphoma (FL1–2, FL3B first, FL3B second) and diffuse large B-cell lymphoma (GCB, ABC and Unclassified DLBCL) samples to investigate their differentiation states, cellular origins and lineage-defining transcriptional programs.

Only tumour cells, previously identified using clonal and CNA-based criteria, were included. Cells with extreme values for the number of detected genes, total UMI counts or mitochondrial transcript fractions were removed. To avoid technical and clonal biases in clustering, mitochondrial, ribosomal and immunoglobulin genes (*IGH, IGK, IGL, IGHD*) were excluded from the feature set. Gene expression values were normalized and log-transformed (normalize_total, log1p). We selected 2,500 highly variable genes, scaled the data and performed principal component analysis (PCA). Batch effects across samples were reduced using Harmony^96^, after which a nearest-neighbour graph was constructed and clusters were identified with the Leiden algorithm. Data were visualized using UMAP. As previously described, clusters were manually annotated based on the expression profiles of canonical B-cell marker genes. Further refinement, guided by differential gene ranking between these clusters, facilitated the definition of germinal-center-related differentiation states.

RNA velocity was estimated using scVelo^97^, and cell-state transitions were modelled with CellRank. The VelocityKernel and ConnectivityKernel were combined, and the GPCCA estimator was used to identify macrostates (n = 6). Terminal states were defined as those with the highest stationary probability in the coarse-grained transition matrix. Fate probabilities were computed for all cells and visualized across clusters.

Pseudotime trajectories were inferred using Palantir^98^, based on the previously defined initial and terminal states. Pseudotime distributions across clusters were examined to characterize differentiation progression. Lineage-specific driver genes were identified with CellRank (compute_lineage_drivers), and genes with p < 0.05 were considered significant. Gene expression dynamics along pseudotime were visualized using heatmaps.

### Differential Gene Expression (DEG) and Gene Set Enrichment Analysis (GSEA)

Differential expression at the single-cell level was performed with the muscat package in R^99^. The integrated .h5ad dataset was imported into Seurat and converted to a SingleCellExperiment object while retaining metadata and UMAP coordinates. Only malignant B cells were included in the analysis, with Normal sorted GC B cells used as the reference group. Comparisons were performed for FL3B versus other lymphoma subtypes, FL1–2 versus other subtypes, and between the FL3B first and FL3B second groups.

Low-quality cells and lowly expressed genes were removed. Library-size normalization and log1p transformation were applied. Cell-type labels, group labels, and sample identifiers were assigned using prepSCE^99^, and clusters absent in one of the groups were excluded. Group sizes within each B-cell subtype were balanced by downsampling. DEG testing was then carried out using muscat’s mmDS^99^ workflow with the dream mixed-model framework. Genes with FDR < 0.05 and ∣log_2_FC∣ > 1 were considered differentially expressed. As an independent validation, pseudobulk differential expression was performed. All malignant B cells were treated as a single cluster, and sample-level pseudobulk profiles were generated using aggregateData^99^. After filtering and normalization, differential expression was computed with pbDS^99^. Genes with ∣log_2_FC∣ > 1 and FDR < 0.05 were considered significant.

Genes were ranked by log_2__fold change, and enrichment of Reactome pathways^100^ was assessed using the standard GSEA framework implemented in the clusterProfiler package^101^ (RRID:SCR_016884). Pathways with FDR < 0.05 were considered significant, and enrichment direction was determined from the normalized enrichment score (NES). Summary tables list the top three enriched pathways for each comparison.

### TME analysis

#### Assembly and initial filtering of the unified TME cohort

To further investigate the TME, we assembled a dataset comprising 48 samples: FL1-2 (n = 21), FL3B (n = 10; subdivided into FL3B-a, n = 4, and FL3B-b, n = 6), GCB DLBCL (n = 8), ABC DLBCL (n = 3), unclassified DLBCL (n = 2), and normal lymph nodes (n = 4 samples obtained from 2 individuals).

The final TME cohort was generated by merging non-malignant, non-B-cell populations from two independently processed cohorts. In the lymphoma cohort, TME cells were extracted from a previously established global single-cell object (see Sample Integration into a Unified Single-Cell Object). In the normal control cohort, normal lymph node samples were processed the same quality control and empty droplet filtering procedures applied to the lymphoma cohort (see Quality Control). Samples from both cohorts were integrated using the Harmony algorithm, followed by clustering with the Leiden algorithm. Major immune lineages were annotated based on cluster-specific marker gene expression. B-cell exclusion was performed in two sequential steps in both cohorts. First, Leiden clusters with high expression of canonical B cell markers (*MS4A1, CD79A*, and *CD19*) were identified through cluster-level marker analysis and removed. Then, residual B-cell contamination was eliminated at the single-cell level by excluding cells with \ >1 UMI for any of these three markers. The completeness of B-cell removal was assessed by examining the residual expression of immunoglobulin genes (*IGH, IGK*, and *IGL* gene families) and plasmablast-associated markers (*MZB1, JCHAIN, SDC1*, and *TNFRSF17*), followed by visual inspection of UMAP embeddings. The purified TME subsets from both cohorts were concatenated into a unified AnnData object and subjected to an additional round of quality control filtering. Cells were excluded if they expressed < 200 or > 3,800 genes or if the fraction of mitochondrial transcripts exceeded 5%.

### Batch-effect correction, clustering, and annotation

To reduce technical and stress-related sources of variation, mitochondrial genes (MT-), ribosomal genes (RPS and RPL), and the long non-coding RNA *MALAT1* were excluded from the expression matrix. Gene expression counts were normalized to 10,000 counts per cell and log-transformed using log1p. The top 2,000 highly variable genes were selected using patient ID as a batch key to account for inter-patient variability during feature selection. The expression matrix was subsequently scaled with a maximum value of 10, followed by PCA. Batch effects across the 48 samples were corrected using the Harmony algorithm^102^ applied to the PCA embedding via scanpy.external.pp.harmony_integrate, using patient ID as the integration covariate and default parameters. A KNN graph was then constructed from the Harmony-corrected embedding using the 35 neighbors. Unsupervised clustering was performed with the Leiden algorithm with a resolution of 0.6. Partition-based graph abstraction (PAGA) was applied to the Leiden clusters, and the resulting graph structure was used to initialize UMAP embedding (minimum distance = 0.3).

Cluster-specific marker genes were identified using the Wilcoxon rank-sum test implemented in the rank_genes_groups function in Scanpy, with p-values adjusted using the Benjamini-Hochberg procedure. Cell type annotation was performed by manually integrating cluster-specific differential expression profiles with canonical marker gene signatures, using by Azimuth Human Tonsil v2 Reference Atlas^103^. The initial 14 Leiden clusters were consolidated into 11 TME cell types, including CD4^+^ and CD8^+^ T-cell subsets, NK cells, cycling T cells, plasmacytoid dendritic cells, and macrophages.

### Comparative analysis of TME cell subtype proportions via permutation testing

Differences in the proportions of TME cell subtypes between diagnostic groups were assessed using a permutation-based approach implemented in scProportionTest. For each pairwise comparison, diagnostic labels were randomly permuted across cells, and subtype proportions were recalculated for each permutation. The observed differences in proportions were then compared against the resulting empirical null distribution to derive test statistics and significance values. A total of 1,000 permutations were performed were performed for each comparison.

### DEG analysis of TME populations

DEG analysis was performed to identify transcriptional alterations in TME cell populations across lymphoma subtypes relative to normal lymph nodes. A pseudobulk strategy was applied when sufficient biological representation was available. The annotated AnnData object was imported into R, converted to a Seurat object, and subsequently transformed into a SingleCellExperiment object to enable analysis with the muscat framework.^99^ To avoid pseudoreplication, technical replicates from the same donor in the normal lymph node cohort were merged under a single biological sample identifier prior to analysis.

Raw UMI counts were aggregated per sample and cell type using the aggregateData function from the muscat package with sum-based aggregation. Sample-cell type combinations represented by fewer than 10 cells were excluded. Differential expression testing was performed independently for each cell type, comparing each lymphoma subtype to normal lymph nodes using the pbDS function with the edgeR method. P-values were adjusted using the Benjamini-Hochberg procedure.

For ABC DLBCL and unclassified DLBCL, the pseudobulk analysis was not feasible due to insufficient biological sample representation across cell types. For these groups, cell-level DEG analysis was performed in Scanpy^80^ using the Wilcoxon rank-sum test with Bonferroni correction. To mitigate the effects of group size imbalance, cells were randomly downsampled to match the smaller group, with a maximum of 1,000 cells per condition. Cell type–subtype combinations with fewer than 20 cells in either group were excluded. These cell-level analyses are considered exploratory due to the limited biological replication.

### GSEA of TME populations

GSEA was performed in Python (RRID:SCR_024202) using the gseapy.prerank^104^ function. For each cell type-contrast pair, a ranked gene list was generated from DEG results, with genes ordered by log_2_ fold change. For cell-level DEG analyses, log_2_ fold change values were clipped to the range [− 10, 10] to limit the influence of extreme outliers on enrichment scores. Duplicate gene symbols and entries with missing values were removed prior to analysis. Pre-ranked gene lists were indipendetly tested against three curated gene sets collections: Gene Ontology (GO) Biological Process (MSigDB, 2025)^25^, Reactome pathways (2026)^100^, and MSigDB Hallmark gene sets (v2026.1)^105^. Enrichment scores were calculated using 1,000 permutations with a fized random seed (seed = 42). For visualization, housekeeping-related pathways (e.g., ribosome biogenesis, RNA splicing, and translation) and pathways not directly related to hematopoietic biology (e.g., muscle, neuronal, and epithelial programs) were excluded from downstream plots. These exclusions were applied solely for visualization purposes and did not affect the underlying enrichment statistics.

### Cell-cell communication analysis

To investigate intercellular communication between malignant B cells and TME populations, cell-cell communication analysis was performed in R using CellChat (v2)^44^ with the CellChatDB v2 database. Cell type annotations derived from the TME analysis were transferred to the global single-cell object by barcode matching. The annotated object was log-normalized and stratified by diagnosis, and a separate CellChat object was created for each diagnostic group.

Within each group, overexpressed genes for each cell type were identified using the identifyOverExpressedGenes function, followed by identification of overexpressed ligand-receptor interactions using identifyOverExpressedInteractions. Communication probabilities were computed using computeCommunProb, excludig interactions involving cell groups with fewer than 10 cells. pathway-level communication probabilities were then calculated using computeCommunProbPathway. Only interactions with CellChat-derived p-values < 0.05 were retained.

For downstream analysis, interactions were restricted to those in which malignant B cells served as either the source or target population. These filtered interactions were exported for visualization using bubble plots.

#### Mass Cytometry

Cytometry by time-of-fight (CyTOF) was performed on tumor samples from 90 FL lymph node biopsies (FL1-2 n = 62, FL3A n = 14, FL3B n = 14), as previously described^106^. Briefly, 3 × 10^6^ cells were stained using 5 μmol/L Cell-ID Cisplatin (Fluidigm) for 5 minutes and quenched with Maxpar Cell Staining Buffer (Fluidigm) at 5 times the volume of the cell suspension. After centrifugation, cell suspensions (50 μL) were incubated with 5 μL of Human Fc Receptor Blocking Solution (BioLegend) for 10 minutes, followed by incubation with 50 μL of a premixed antibody cocktail for 30 minutes (**Suppl Table S10**). All antibodies were purchased from Fluidigm. After washing, cells were incubated overnight at 4 ° C with 1 mL of cell intercalation solution (125 nmol/L Maxpar Intercalator-Ir in 1 mL Maxpar Fix and Pem Buffer; Fluidigm). Cells were then washed with MaxPal Water, pelleted, and resuspended in EQ Calibration Beads (Fluidigm). Data acquisition was performed on a CyTOF II instrument (Fluidigm). CyTOF data were analyzed using Cytobank software (RRID:SCR_014043). All samples were normalized and analyzed simultaneously to account for signal variability across extended acquisition times. Linage markers (CD4, CD8, TIM3, PD1, CXCR5, CCR7, CD45RO, ICOS, TIGIT, or BTLA) were used to classify T cells into regulatory, naïve, follicular helper, memory, effector, or exhausted subsets. SIRPα and CD14 were used to identify monocytes and macrophages.

#### Clinical Statistical Analysis

Comparisons of baseline clinical characteristics between subgroups were performed using two-sample *t* tests, one-way analysis of variance (ANOVA), or Pearson's χ^2^ tests, as appropriate based on the data type and study design. All tests were two-sided. Effect estimates are reported with corresponding 95% confidence intervals (CIs), and *P* values are presented without adjustment for multiple comparisons unless otherwise specified. Statistical significance was defined as a two-sided *P* < 0.05.

EFS was defined as the time from diagnosis to disease progression, relapse, retreatment, or death from any cause. OS was defined as the time from diagnosis to death from any cause. The cumulative incidence of histologic transformation was analyzed using a competing-risks framework, treating progression or retreatment of FL without transformation and death before transformation as competing events. Cumulative incidence functions were estimated and compared according to TME subgroup. Statistical analyses were performed in R (v4.2.2; RRID:SCR_001905).

## Supplementary Material

This is a list of supplementary files associated with this preprint. Click to download.

- SupplTableS7.CNVlist.xls
- SupplTableS8.PhenoClerpanel.docx
- SupplTableS10.CytoFpanel.docx
- SupplTableS1.TMEanalysisinFL.docx
- SupplTableS3.PatientCharacteristicsTMEFL.docx
- SupplTableS5.PatientCharacteristicsTMEDLBCL.docx
- SupplTableS2.TMEanalysisinDLBCL.docx
- SupplTableS6.Markergenesforsinglecell.docx
- SUPPLEMENTARYFIGURELEGEND.docx
- SupplementaryFigures.pdf
- SupplTableS4.Competingriskatfirstevent.docx
- SupplTableS9.Spatialcommunityclustersidentification.docx

## Data Availability

scRNA-seq and scBCR-seq data of FL3B, DLBCL and healthy donors have been deposited in NCBI GEO via GSE337922. Previously publishedscRNA-seq and scBCR-seq data ofnewly diagnosed patients with FL1-3A are deposited in NCBI GEO via GSE290351^7^. WES and RNA-seq data of newly diagnosed patients with FL and DLBCL are available in dbGaP via accession phs002989.v1.p1^18^ and phs003634v1^20^.
